# Amphiphilic Macromolecular Dendritic Antioxidants with Surfaces Coated in Hybrid Phenolic Units That Provide Anti-Inflammatory Properties

**DOI:** 10.1021/acsapm.5c04280

**Published:** 2026-02-20

**Authors:** Blessed Agbemade, Fati Haruna, Aundrea Stengard, Nanzhu Li, Cal M. Butts, Rebecca Uzarski, Choon Young Lee

**Affiliations:** † Department of Chemistry and Biochemistry, 5649 Central Michigan University, Mount Pleasant, Michigan 48859, United States; ‡ Science of Advanced Materials Program, 5649 Central Michigan University, Mount Pleasant, Michigan 48859, United States; § Department of Biology, 5649 Central Michigan University, Mount Pleasant, Michigan 48859, United States

**Keywords:** antioxidant dendrimers, syringaldehyde, pyridoxal, DPPH assay, anti-inflammatory, nitric oxide, interleukin-6, cytotoxicity

## Abstract

Excess free radicals cause oxidative stress, which damages cells and triggers inflammation. Inflammation generates more radicals, creating a self-perpetuating cycle that contributes to many human diseases. Antioxidants can neutralize radicals and prevent inflammation. Two unique amphiphilic dendritic antioxidants, Generation 1 (G1) and Generation 2 (G2), were developed, each featuring an equal ratio of hydrophobic syringaldehyde to water-soluble pyridoxal on their surfaces. G1 carries six units of each component, whereas G2 contains 12 units of each. In the 2,2-diphenyl-1-picrylhydrazyl radical-scavenging assay, G2 and G1 exhibited IC50 values of 1.28 and 2.6 μM, respectively. In comparison, syringaldehyde and pyridoxal exhibited significantly higher IC50 values of 200 and 16500 μM, respectively. G2 and G1 are 156 and 77 times more effective than syringaldehyde and 12890 and 6346 times more potent than pyridoxal, highlighting the benefits of building antioxidants within dendritic frameworks. In cell viability assays with RAW 264.7 macrophages, G1 only reduced cell viability at the highest tested concentration (323 μM), while G2 induced a statistically significant decrease starting at 11.3 μM. In lipopolysaccharide-stimulated macrophages, G1 at 32.3 μM showed strong anti-inflammatory effects, lowering NO levels by 76% and IL-6 levels by 100%, indicating that G1 can produce its potent anti-inflammatory effects via dual mechanisms: scavenging reactive oxygen species and reducing pro-inflammatory cytokine production. G2 had limited effects at its nontoxic concentrations.

## Graphical abstract

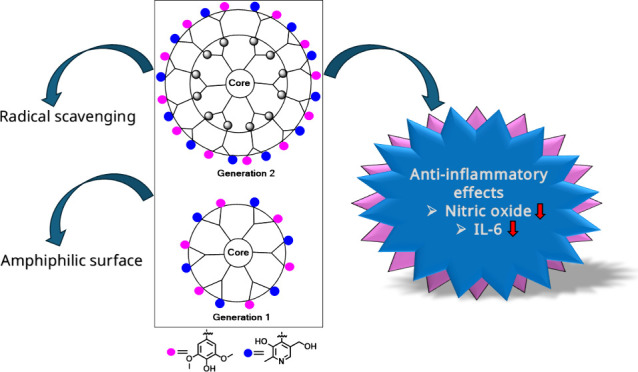

## 1 Introduction

It is well-known that free radicals, when produced in excess, disrupt cellular balance and cause oxidative stress. This imbalance damages cellular components, including lipids, proteins, and DNA, impairing cellular function and activating signaling pathways that lead to inflammation. Inflammation can further amplify oxidative stress because activated immune cells, such as macrophages, generate reactive oxygen and nitrogen species (ROS and RNS), commonly referred to as free radicals, to fight pathogens. This creates a vicious cycle in which oxidative stress and inflammation mutually reinforce one another. Chronic oxidative stress and inflammation may lead to the onset and progression of many human diseases, including cardiovascular disorders, neurodegeneration, diabetes, and cancer.

Antioxidants can reduce oxidative stress and prevent inflammation. They are available in various forms with different mechanisms of action, including radical quenchers, metal chelators, inhibitors of oxidant-generating enzymes, upregulation of endogenous antioxidant enzymes, and regeneration of other antioxidants.^1^ Among antioxidants, phenol-based antioxidants are the most ubiquitous, and most of these phenolic antioxidants work by neutralizing free radicals. The key features of highly effective radical-scavenging antioxidants include electron-donating groups (EDGs) located ortho/para to the phenolic OH and extensive conjugation, as these properties can stabilize the radicals formed on antioxidants after their hydrogen atom donation to free radicals (radical scavenging).^2–5^ Studies show that the NH_2_ group ortho/para to the phenolic OH is the most efficient EDG and beneficial for antioxidant effects, followed by the OH group and then the methoxy group.^6,7^ Considering the fact that phenolic antioxidants with amino groups are quite rare in nature, phenolic antioxidants with consecutive OH groups, such as catechol or galloyl groups, are the strongest antioxidants. However, unfortunately, they exhibit pro-oxidant effects when exposed to transition metal ions like copper or iron, due to their ability to effectively complex with transition metal ions.^8–12^ In our previous study, small *o*-methoxy-substituted antioxidants, such as syringaldehyde (with two *o*-methoxy groups) or vanillin (with one *o*-methoxy group), were assembled into dendritic structures, which led to strong antioxidant effects without causing pro-oxidant effects.^13,14^ Among the dendrimers, syringaldehyde-based dendrimers consistently outperformed their vanillin counterparts, exhibiting superior antioxidant activities without inducing pro-oxidant effects, indicating the importance of *o*-methoxy groups. However, multiple syringic units on the surface of antioxidant dendrimers increase hydrophobicity, thereby limiting their use in biomedical applications. The major challenge with antioxidants is that potent hydrophilic antioxidants often cause pro-oxidant effects, whereas effective antioxidants that lack pro-oxidant activity tend to be hydrophobic. In developing antioxidants for medical use, the most important properties include strong radical-scavenging ability, no pro-oxidant effects, and good solubility in biological fluids. Therefore, enhancing the solubility of antioxidant dendrimers while preserving their beneficial properties is imperative for optimizing their efficacy in medical applications.

Syringaldehyde is a naturally occurring molecule known to exhibit not only antioxidant activity but also a range of promising pharmacological effects,^15^ including antimicrobial,^16^ antihyperglycemic,^17,18^ anti-inflammatory,^19–21^ and anticancer properties^22^ as well as COX-2 inhibition.^23^ Although incorporating syringaldehyde into dendritic frameworks increases hydrophobicity, it remains a valuable building block for developing antioxidant dendrimers for medical use due to its broad therapeutic potential and its ability to provide strong antioxidant protection without causing pro-oxidant effects.

Macromolecular antioxidants, also known as nanoantioxidants, employ nanotechnology to mitigate oxidative stress. They mostly refer to nanomaterials or particles that exhibit antioxidant properties, either inherently or by functionalizing particles with antioxidant molecules. These nanoclass antioxidants are typically created by encapsulating antioxidants within the cavities of polymers or dendrimers,^24–26^ or by conjugating small antioxidants to their surfaces.^27–29^ These constructs are designed to improve antioxidant efficacy and to address the limitations associated with small antioxidant molecules. These macromolecular antioxidants are reported to have better solubility and stability compared to natural antioxidants and protect antioxidant molecules from early degradation and metabolism, enhancing their effectiveness.^30–32^ One of the key benefits is their ability to perform multiple functions. For example, they can serve as carriers for other molecules, such as drugs, combining their inherent antioxidant properties with therapeutic effects.^30,33^ Additionally, their size and surface composition can be tailored to optimize antioxidant activity and compatibility with biological systems.^33^

In the current study, two unique dendritic antioxidants were designed to incorporate multiple syringaldehyde units, along with an equal number of water-soluble pyridoxal units, on the dendrimer surface with the intention of improving the solubility profiles of syringaldehyde-based antioxidant dendrimers. This report delineates the synthesis of these antioxidant dendrimers, as well as their DPPH radical-scavenging activity, cell viability, and anti-inflammatory properties.

## 2 Materials and Methods

### 2.1 Chemicals

d-Mannitol, syringaldehyde, pyridoxal–HCl, sodium triacetoxyborohydride [NaBH­(OAc)_3_], 2-picoline borane, sodium azide, *p*-toluenesulfonyl chloride, sodium hydride in powder form, triethylamine (TEA), 2,2-diphenyl-1-picrylhydrazyl (DPPH), and ethylenediaminetetraacetic acid (EDTA) disodium salt dihydrate were purchased from Sigma-Aldrich (Milwaukee, WI, USA). 2-(2-Chloroethoxy)­ethanol and 2-[2-(2-chloroethoxy)­ethoxy]­ethanol were obtained from AmBeed Inc. (Buffalo Grove, IL, USA). Copper metal granules (99.9%, 100 mesh) were obtained from Atlantic Equipment Engineers (Upper Saddle River, NJ, USA). All chemicals were used as received without further purification. All reaction solvents, as well as deuterated NMR solvents, were purchased from VWR (Visalia, CA, USA). Liquid chromatography/mass spectrometry (LC/MS)-grade acetonitrile and formic acid were obtained from Honeywell (Lodi, NJ, USA) and ThermoFisher Scientific (Waltham, MA, USA), respectively. Ultrapure water was obtained from our Milli-Q UVPlus unit (Millipore, France). The Sephadex LH-20 column was purchased from iPure Biology Co., Ltd. (Hong Kong, China). Cell lines were purchased from ATCC (Manassas, VA, USA). Cell culture reagents were purchased from Sigma-Aldrich (St. Louis, MO, USA). The Invitrogen enzyme-linked immunosorbent assay kit (88-7064-22) for interleukin-6 (IL-6) was purchased from ThermoFisher Scientific (Waltham, MA, USA).

### 2.2 General Information for Synthesis and Purification

Copper-catalyzed alkyne–azide click reactions were conducted in a microwave reactor (CEM, Discover 2.0). Copper granules (99.99%, 100 mesh) were soaked in a 20% sodium hydroxide solution for 30 min, subsequently rinsed with water, then immersed in a 20% sulfuric acid solution for 30 min, rinsed again with water, followed by an acetone rinse, dried, and stored under an argon atmosphere.

For purification, either silica gel column chromatography using a commercially available prepacked silica gel column (40 or 120 g, 230–400 mesh, Luknova, Mansfield, MA, USA) on the CombiFlash Companion (Teledyne Isco) system or size-exclusion chromatography with a Sephadex LH-20 (50 g) column was employed.

Fractions containing the target compounds were analyzed using a standalone HPLC system (Hitachi, Japan) to assess purity and combine similar fractions. The mobile phase was a gradient of acetonitrile and water, ranging from 5% to 95% acetonitrile with 0.1% trifluoroacetic acid. The flow rate was 1 mL/min, and analytes were detected at 214 nm.

### 2.3 Characterization

^1^H NMR spectra were recorded on a 500 MHz NMR spectrometer (Bruker, Billerica, MA, USA). Chemical shifts (δ) are reported in ppm. The NMR solvent for each compound was indicated in the synthesis section. The sample concentration was 20 mg/mL. Coupling constants (*J*) are given in Hz. The multiplicities of signals are denoted as follows: s = singlet, br.s = broad singlet, t = triplet, dd = doublet of doublets, dtd = doublet of triplet of doublets, t+t = triplet overlapped with another triplet, and m = multiplet. ^13^C NMR spectra were recorded on a 125 MHz NMR (Bruker, Billerica, MA, USA).

Mass spectra were obtained using either an ultraperformance liquid chromatography (UPLC)/electrospray ionization (ESI)-quadrupole time-of-flight (Q-TOF) mass spectrometer (AdvanceBio 6545XT, Agilent, Santa Clara, CA, USA) or a high-performance liquid chromatography (HPLC)/electrospray ionization (ESI)-time-of-flight (TOF) mass spectrometer (G6230B, Agilent, Santa Clara, CA, USA). Both the UPLC and HPLC systems are equipped with a multiwavelength diode array detector (Agilent, Santa Clara, CA, USA).

The LC/MS samples were prepared at a concentration of 1 ng/mL. For fractions obtained from column chromatography, samples were prepared by performing three serial dilutions in the matrix (water–acetonitrile, 50:50, with 0.1% formic acid). Prior to injection, all samples were filtered through a syringe filter (Minisart RC4, cellulose membrane, 0.2 μm pore size; Sartorius, Epsom, U.K.). The injection volume was 0.3 μL. LC separations were conducted on an Agilent C18 column (InfinityLab Poroshell 120 EC-C18 with a mean particle size of 1.9 μm, 2.1 mm inner diameter, and 50 mm length) utilizing a water–acetonitrile gradient system (from 5% to 95% acetonitrile) containing 0.1% formic acid. The flow rate was 0.4 mL/min for 10 min, with the column oven temperature kept at 35 °C. Mass analysis of all samples was performed using Dual Agilent Jet Stream Electrospray Ionization (AJS ESI) as the ion source under these conditions: gas temperature at 320 °C, drying gas flow rate at 8 L/min, nebulizer gas at 35 psi, sheath gas temperature at 350 °C, sheath gas flow at 11 L/min, capillary voltage at 3500 V, nozzle voltage at 1000 V, fragmentor voltage at 120 V, skimmer voltage at 65 V, MS range from *m*/*z* 100 to 3200, and an acquisition rate of 1 spectrum per second. Tuning and calibration of the instrument were performed using an ESI-L low-concentration tuning mix and hexamethoxyphosphazine (0.1 mM HP-0321), both purchased from Agilent.

### 2.4 Software

The ChemDraw programs were used to draw the structures of the compounds. The NMR data were processed using the MNova NMR program (version 16.0). To help assign ^1^H and ^13^C NMR signals, the MNova NMRPredict program (version 16.0) was used.

### 2.5 Dynamic Light Scattering (DLS) Spectroscopy

The hydrodynamic diameter of each dendrimer was determined using the Malvern Zetasizer Nano ZS (Malvern Panalytical, Malvern, U.K.), a DLS instrument. Sample solutions of G1 at 10 mg/mL and G2 at 20 mg/mL were prepared in *N*,*N*-dimethylformamide (DMF) and filtered through a 0.22 μm Teflon syringe filter. Measurements were performed at 25 °C using a PCS1115 glass cuvette with a path length of 10 mm. The instrument was set to operate at a scattering angle of 173° (backscatter detection) and a wavelength of 633 nm. Each sample was equilibrated for 2 min before data acquisition. For each measurement, at least three runs were performed, and the average hydrodynamic diameter and polydispersity index (PDI) were calculated using cumulant analysis. The viscosity and refractive index of the dispersant were set based on the properties of the DMF at 25 °C: viscosity = 0.802 cP and refractive index = 1.43.

### 2.6 DPPH Assay

Antioxidant activity was assessed using standardized methods with minor modifications.^34^ DPPH solution was prepared in methanol at 0.0897 mM. All antioxidants were dissolved in 5% PEG600 in methanol. The concentrations of antioxidants were prepared at 0.15–0.002344 mM for compound **10** (G2), 0.3–0.004688 mM for compound **7** (G1), 1.00–0.015625 mM for compound **4** (surface building block), 50–0.78125 mM for syringaldehyde, and 1000–15.625 mM for pyridoxal–HCl. A volume of 25 μL of antioxidant solution or 5% PEG in methanol (used as blank) was added to 1.2 mL of DPPH reagent. The final antioxidant concentrations ranged from 3.125 to 0.0488 μM for compound **10** (G2), from 6.25 to 0.049 μM for compound **7** (G1), from 20.83 to 0.3255 μM for compound **4**, from 1041.67 to 16.27 μM for syringaldehyde, and from 20833.33 to 325.52 μM for pyridoxal HCl. Samples were incubated at room temperature in the dark for 1 h before absorbance was measured at 515 nm. All experiments were performed in triplicate, with the coefficient of variation for percent inhibition remaining below 6%. The IC50 value of each antioxidant was determined from its graph, which plotted the concentration against % DPPH remaining, calculated as 100 – {[(absorbance of blank – absorbance of sample)/absorbance of blank] × 100}.

### 2.7 Cell Viability Assay

RAW 264.7 murine macrophage cells were cultured in RPMI 1640 media supplemented with 10% fetal bovine serum (FBS) and 2 mM glutamine at 37 °C, in a 5% CO_2_-humidified incubator. To assess viability, 100 μL of cells (5 × 10^5^/mL) was added to 96-well plates and incubated with 100 μL media control or test compound at varying concentrations for 24 h. A volume of 20 μL of a 5 mg/mL 3-(4,5-dimethylthiazole-2-yl)-2,5-diphenyltetrazolium bromide (MTT) solution in 0.01 M phosphate-buffered saline was added to each well 2 h before termination of the experiment. The plates were centrifuged (450*g*, 10 min), and supernatants were removed. The resulting formazan crystals were dissolved in 100 μL of dimethyl sulfoxide (DMSO), and absorbance was measured using a Biolog microplate reader (Biotek Instruments) at dual wavelengths of 590 and 650 nm. Percent control response was calculated (absorbance of treatment/absorbance of control × 100). All experiments were performed in triplicate and repeated.

### 2.8 Anti-inflammatory Activity Assay

Cells were cultured in RPMI 1640 without phenol red, supplemented with 10% FBS and 2 mM l-glutamine. To assess inflammatory mediators, 500 μL of cells (1 × 10^6^ cells/mL), media (control), or test compound at varying concentrations was added to 6-well plates to a volume of 975 μL and incubated for 6 h at 37 °C, under 5% CO_2_. Each well was treated with 5 μg of bacterial lipopolysaccharide (LPS; *Escherichia coli* O55:B5) for 24 h. Supernatants were immediately analyzed for nitrite or frozen for IL-6 analysis.

### 2.9 Nitric Oxide (NO) Assay

A NO assay was used to determine the nitrite concentration as an indicator of NO production. Supernatants (100 μL) were combined with 100 μL of Griess reagent (0.1% *N*-1-naphthylethylenediamine dihydrochloride and 1% sulfanilamide in 5% phosphoric acid) in 96-well plates, incubated for 10 min at room temperature. Absorbance was recorded at 590 nm. Percent control response was calculated (absorbance of treatment/absorbance of control × 100). All experiments were performed in triplicate and repeated.

### 2.10 IL-6 Analysis

IL-6 levels were measured using an Invitrogen enzyme-linked immunosorbent assay kit according to the manufacturer’s instructions.

### 2.11 Synthesis Methods and Analysis Results

#### Compound **1**

2-[2-(2-Chloroethoxy)­ethoxy]­ethanol (10 mL, 1.16 g/mL, 168.62 g/mol, 68.7937 mmol), toluene (50 mL), and TEA (14 mL, 0.726 g/mL, 101.19 g/mol, 100.4447 mmol) were added to a 500 mL three-neck flask in an ice bath and stirred for 5 min. Then, *p*-toluenesulfonyl chloride (15.7386 g, 190.65 g/mol, 82.5523 mmol) was dissolved in 150 mL of toluene and transferred to a separatory funnel. The solution was added dropwise over 30 min. Upon completion of the addition, the solution was stirred for 1 h, and the ice bath was removed. The reaction was run for 12 h and then was worked up into toluene and water layers. Magnesium sulfate was added to the toluene layer, the mixture was filtered, and the filtrate was then rotary-evaporated. The resulting mixture was loaded neat onto a 120 g silica gel column pretreated with hexane–TEA (200:1), followed by hexane. It was purified using a hexane–ethyl acetate gradient system (10:1 to 5:1), and the target compound eluted at 6:1 hexane–ethyl acetate. The pure fractions were combined based on thin-layer chromatography (TLC) and LC/MS, rotary-evaporated, and kept under house vacuum overnight before analysis.

Yield = 85% (18.77 g); *R*_*f*_ = 0.47 in hexane–acetone (1:1); appearance = colorless oily material. ^1^H NMR (500 MHz, CDCl_3_): δ 7.29 (d, *J* = 8.4 Hz, 2H), 6.86 (d, *J* = 8.2 Hz, 2H), 3.69–3.64 (m, 2H), 3.20 (dt, *J* = 9.2 and 5.1 Hz, 4H), 3.13–3.04 (m, 6H), 1.94 (s, 3H). ^13^C NMR (126 MHz, CDCl_3_): δ 144.88, 132.94, 129.87, 127.82, 71.21, 70.53, 70.40, 69.40, 68.58, 42.91, 21.50. LC/ESI-TOF MS. Calcd for C_13_H_19_ClNaO_5_S ([M + Na]^+^): *m*/*z* 345.0534. Found: *m*/*z* 345.0560.

#### Compound **2**

4-Hydroxybenzaldehyde (4.5388 g, 122.12 g/mol, 37.1667 mmol) was weighed and transferred into a 250 mL flask. DMF (100 mL) was added, and the mixture was stirred until complete dissolution. Potassium carbonate (8.5611 g, 138.21 g/mol, 61.9427 mmol) was added and stirred for 30 min to 1 h at 60–70 °C. Compound **1** (13.3300 g, 322.0642 g/mol, 41.3893 mmol) was dissolved in 30–50 mL of DMF and added to the reaction mixture. This reaction was left to run for 14 h. LC/MS confirmed that the reaction was complete. The mixture was filtered and then rotary-evaporated, and the product was extracted with ethyl acetate and water. Anhydrous magnesium sulfate was added to the ethyl acetate layer and then filtered off. The filtrate was concentrated on a rotary evaporator. The resulting residue was dissolved in acetone and combined with silica gel to form a slurry. The dried slurry was loaded onto a 120 g silica gel column, which was pretreated with hexane–TEA (200:1) and then with hexane. A hexane–ethyl acetate gradient (100:0 to 50:50) on an automated system (CombiFlash Companion) was used for purification, eluting the compound at 60:40. Fractions containing the target compound were combined, rotary-evaporated, and kept under vacuum overnight for analysis and use.

Yield = 80% (8.05 g); *R*_*f*_ = 0.46 in hexane–acetone (1:1); appearance = white solid. ^1^H NMR (500 MHz, CDCl_3_): δ 9.81 (s, 1H), 7.76 (d, *J* = 8.8 Hz, 2H), 6.96 (m, *J* = 8.8 Hz, 2H), 4.18–4.13 (m, 2H), 3.72–3.65 (m, 2H), 3.66–3.61 (m, 6H), 3.56 (t, *J* = 5.9 Hz, 2H). ^13^C NMR (126 MHz, CDCl_3_): δ 190.93, 164.04, 132.10, 130.26, 115.10, 71.57, 71.04, 70.86, 69.71, 67.98, 42.98. LC/ESI-TOF MS. Calcd for C_13_H_18_ClO_4_ ([M + H]^+^): *m*/*z* 273.0888. Found: *m*/*z* 273.0936.

#### Compound **3** (Internal Building Block)

Compound **2** (7.0300 g, 272.0815 g/mol, 25.8378 mmol) was weighed and transferred into a 250 mL round-bottomed flask. 1,2-Dichloroethane (150 mL) was added with continuous stirring to dissolve compound **2**. Propargylamine (0.75 mL, 0.86 g/mL, 55.08 g/mol, 11.7102 mmol) was added and left to react for 1 h. NaBH­(OAc)_3_ (7.4497 g, 211.94 g/mol, 35.1500 mmol) was added. The reaction was complete in 4 h. The mixture was rotary-evaporated and partitioned into ethyl acetate and water layers. Magnesium sulfate was added to the ethyl acetate layer containing the product, and the mixture was then filtered. The filtrate was concentrated on a rotary evaporator. The resulting residue was dissolved in acetone and mixed with silica gel to make a slurry. The dried slurry was then loaded onto a 120 g silica gel column, which was pretreated with hexane–TEA (200:1), followed by hexane. The purification was performed using a CombiFlash Companion with a hexane–ethyl acetate gradient (100:0 to 50:50). The target compound eluted at 60:40 hexane–ethyl acetate. The fractions containing the target compound were combined, dried by rotary evaporation, and kept under vacuum overnight before analysis.

Yield = 77% (5.10 g); *R*_*f*_ = 0.40 in hexane–ethyl acetate (1:1); appearance = colorless oily material. ^1^H NMR (500 MHz, CDCl_3_): δ 7.26 (d, *J* = 8.7 Hz, 4H), 6.86 (d, *J* = 8.6 Hz, 4H), 4.10 (dd, *J* = 5.8 and 4.0 Hz, 4H), 3.83 (dd, *J* = 5.8 and 4.1 Hz, 4H), 3.76–3.64 (m, 12H), 3.59 (dd, *J* = 12.2 and 6.3 Hz, 8H), 3.20 (d, *J* = 2.5 Hz, 2H), 2.27 (t, *J* = 2.3 Hz, 1H). ^13^C NMR (126 MHz, CDCl_3_): δ 157.96, 131.11, 130.14, 114.44, 78.65, 73.36, 71.39, 70.80, 70.69, 69.84, 67.46, 56.65, 42.75, 40.79. LC/ESI-TOF MS. Calculated for C_29_H_40_Cl_2_NO_6_ ([M + H]^+^): *m*/*z* 568.2227. Found: *m*/*z* 568.2239.

#### Compound **4** (Surface Building Block)

Pyridoxal–HCl (3.8000 g, 203.62 g/mol, 18.6622 mmol) was added to a 250 mL round-bottomed flask and dissolved in 100 mL of methanol. Propargylamine (1.00 mL, 0.86 g/mL, 55.08 g/mol, 15.6137 mmol) was added, and the reaction mixture turned yellow. After 30 min, 2-picoline borane (1.8000 g, 106.96 g/mol, 16.8287 mmol) was added. The reaction was allowed to proceed overnight at room temperature with continuous stirring. Syringaldehyde (3.3000 g, 182.17 g/mol, 18.1149 mmol) was added to the reaction mixture, and 2-picoline borane (1.8000 g) was introduced with a 30 min interval. The reaction was allowed to run overnight. Reaction progress was monitored by TLC and LC/ESI-TOF MS to confirm the formation of the target compound. The precipitates formed in the reaction were filtered out; LC/ESI-TOF MS analysis confirmed that the target compound was present in the filtrate, not in the residue. The filtrate was then rotary-evaporated and partitioned into ethyl acetate and water layers. Due to the compound’s hybrid nature, approximately 20% migrated to the aqueous layer, while 80% remained in the ethyl acetate layer. The aqueous layer was lyophilized and then extracted with ethyl acetate. Anhydrous magnesium sulfate was added to the organic layer and filtered out, and the filtrate was evaporated using a rotary evaporator. The resulting residue was dissolved in 5 mL of chloroform, and the resulting solution was loaded onto a commercially available 40 g silica gel column and pretreated with hexane–TEA (200 mL:1 mL), followed by 100 mL of hexane. The mixture was purified with a hexane–acetone gradient (3:1 to 1:3). Fractions containing the product were combined, rotary-evaporated, and kept under house vacuum overnight before analysis.

Yield = 26% (1.50 g); appearance = yellowish-white fluffy solid; *R*_*f*_ = 0.214 in hexane–acetone (1:7). ^1^H NMR (500 MHz, CDCl_3_): δ 7.61 (s, 1H), 6.48 (s, 2H), 4.54 (s, 2H), 3.98 (s, 2H), 3.77 (s, 6H), 3.56 (s, 2H), 3.21 (d, *J* = 2.5 Hz, 2H), 2.35 (t, *J* = 2.4 Hz, 1H), 2.31 (s, 3H). ^13^C NMR (126 MHz, CDCl_3_): δ 152.29, 147.39, 146.57, 138.75, 134.81, 132.89, 127.38, 126.62, 106.24, 76.42, 75.41, 60.18, 57.59, 56.17, 51.26, 40.59, 18.13. LC/ESI-TOF MS. Exact mass calculated for C_20_H_25_N_2_O_5_ ([M + H]^+^): *m*/*z* 373.1758. Found: *m*/*z* 373.1758.

#### Compound **5**

The linker was synthesized following the previously published method.^14^

#### Compound **6** (G0.5)

d-Mannitol (1.0000 g, 182.17 g/mol, 5.4894 mmol) was placed into a round-bottomed flask. Anhydrous DMF was then added. After purging the flask with argon, the mixture was heated at 50 °C for 15 min to dissolve d-mannitol. Then, it was cooled to room temperature. Powdered sodium hydride was added, and the mixture was stirred for 1 h. Compound **5** (15.00 g, 285.0783 g/mol, 52.6171 mmol), predissolved in 10 mL of DMF, was added using a cannula needle. The reaction was run for 1 week. The reaction mixture was then filtered through a Celite pad and dried using a rotary evaporator at 50 °C. Then, the residue was dissolved in ethyl acetate (200 mL) and washed with water three times. The organic layer was dried with anhydrous magnesium sulfate and then filtered. The filtrate was dried using a rotary evaporator. The resulting mixture was dissolved in 5 mL of chloroform and loaded onto a prepacked 40 g silica gel column, which had been pretreated with a hexane–TEA (200:1) mixture. Purification was carried out using a hexane–acetone mixture (1:1) containing 1% TEA. Fractions containing the target compound were identified by LC/MS and combined based on the LC/MS results.

Yield = 57% (2.69 g); *R*_*f*_ = 0.53 in hexane–acetone (1:1); appearance = yellowish oil. ^1^H NMR (500 MHz, acetone-*d*_6_): δ 3.91–3.74 (m, 10H), 3.72–3.68 (m, 14H), 3.67–3.57 (m, 20H), 3.41 (q, *J* = 4.8 Hz, 12H). ^13^C NMR (126 MHz, acetone-*d*_6_): δ 79.70, 79.36, 72.67, 71.69, 71.54, 71.52, 71.28, 70.80, 70.74, 70.73, 70.69, 69.66, 51.65, 51.57. LC/ESI-TOF MS. Calcd exact mass for the target C_30_H_56_N_18_O_12_: 860.4325 amu. The target was found as [M + H]^+^, [M + NH_4_]^+^, [M + Na]^+^, and [M + K]^+^, with monoisotopic masses of 861.4431, 878.4692, 883.4243, and 899.3969 amu, respectively. The theoretical monoisotopic masses are 861.4398, 878.4663, 883.4217, and 899.3957 amu, respectively.

#### Compound **7** (G1)

Compounds **6** (0.7000 g, 860.4325 g/mol, 0.8135 mmol), **4** (2.3600 g, 372.1685 g/mol, 6.3412 mmol), copper granules (230 mg), and a stir bar were added to a 35 mL CEM microwave reaction vessel. Then, anhydrous tetrahydrofuran (THF; 15–20 mL) was added via a cannula needle. The reaction was run for 7 h at 77 °C and 150 W under a nitrogen atmosphere. After confirming the formation of the target compound, the mixture was filtered through a Celite pad. Then, the filtrate was treated with saturated aqueous EDTA solution (30 mL) for 1 h. THF was then rotary-evaporated. The resulting mixture containing water was partitioned between chloroform and water. The target compound was found to be present in greater amounts in the chloroform layer than in the water layer. Therefore, the compound was extracted with chloroform three times. The combined chloroform was dried over MgSO_4_, filtered, and concentrated under reduced pressure. The dried residue was redissolved in 5 mL of chloroform, loaded onto a 40 g prepacked silica gel column, and purified using the CombiFlash Companion with a gradient system of hexane–ethyl acetate–methanol (1:0:0 → 0:1:0 → 0:0:1). Purification was also carried out using size-exclusion chromatography. After redissolving the residue in 5 mL of 10% methanol in chloroform, the solution was loaded onto a Sephadex LH-20 column. Purification was performed using 10% methanol in chloroform as the eluent. Based on the LC/MS analysis, fractions containing the target compound were combined and rotary-evaporated. The dried target compound was then analyzed using NMR.

Yield = 67% (1.68 g); appearance = cream-colored flaky solid. ^1^H NMR (500 MHz, MeOD): δ 7.87 (d, *J* = 6.8 Hz, 6H), 7.76 (d, *J* = 1.5 Hz, 6H), 6.59 (d, *J* = 4.2 Hz, 12H), 4.52 (s, 12H), 4.46 (dq, *J* = 10.9 and 5.0 Hz, 12H), 3.92 (d, *J* = 2.7 Hz, 12H), 3.79 (s, 36H), 3.75 (dt, *J* = 18.0 and 4.8 Hz, 24H), 3.55 (s, 22H), 3.45–3.34 (m, 22H), 2.33 (s, 18H). ^13^C NMR (126 MHz, MeOD): δ 153.91, 149.27, 147.32, 143.60, 139.42, 136.35, 134.67, 130.26, 128.45, 126.22, 108.08, 79.43, 79.01, 72.88, 71.73, 71.64, 71.47, 71.35, 70.25, 69.92, 69.49, 60.83, 59.54, 56.90, 52.35, 51.34, 49.85, 18.41. LC/ESI-TOF MS. Calcd exact mass for the target C_150_H_200_N_30_O_42_: 3093.4436 amu. The target had charge states ranging from [M + 2H]^2+^ to [M + 6H]^6+^; deconvoluted to 3093.4482 amu.

#### Compound **8**

Compound **6** (0.4802 g, 860.4325 g/mol, 0.5581 mmol) and compound **3** (2.5314 g, 567.2154 g/mol, 4.4629 mmol), copper granules (200 mg) and a stir bar were added to a 35 mL CEM microwave reaction vessel. Subsequently, anhydrous THF (20 mL) was added through a cannula needle. The reaction was run at 77 °C and 150 W for 10 h. Then, the reaction mixture was filtered through Celite, and the filtrate was treated with saturated EDTA solution (50 mL) for 1 h. Subsequently, THF was removed on a rotary evaporator. Then, chloroform (100 mL) and water (100 mL) were added. The target compound was found in the chloroform layer. Therefore, the compound was extracted with chloroform two more times. The combined chloroform was then dried over MgSO_4_, filtered, and rotary-evaporated. The residue was redissolved in 5 mL of 10% methanol in chloroform, loaded onto an LH-20 size-exclusion column, and purified using 10% methanol in chloroform as the eluent. The fractions containing the target compound were combined based on MS and HPLC analyses and then dried before analysis.

Yield = 75% (1.79 g); *R*_*f*_ = 0.37 in chloroform–methanol (96:4); appearance = light-yellow honey-like material. ^1^H NMR (500 MHz, CDCl_3_): δ 7.51 (s, 6H), 7.24 (d, *J* = 8.7 Hz, 24H), 6.83 (d, *J* = 6.5 Hz, 24H), 4.42 (t, *J* = 5.4 Hz, 12H), 4.08 (tt, *J* = 5.3 and 2.3 Hz, 24H), 3.84–3.55 (m, 158H), 3.47 (s, 42H). ^13^C NMR (126 MHz, CDCl_3_): δ 157.93, 145.20, 131.56, 130.08, 114.51, 78.76, 78.53, 71.73, 71.49, 70.88, 70.79, 70.63, 70.52, 69.95, 69.72, 69.55, 69.50, 68.80, 67.55, 56.68, 50.14, 50.10, 47.71, 42.87. LC/ESI-TOF MS. Calcd exact mass for the target C_204_H_290_Cl_12_N_24_O_48_: 4,263.7252 amu. Found: [M + 2H]^2+^ ∼ [M + 11H]^11+^; deconvoluted to 4263.7503 amu.

#### Compound **9** (G1.5)

Compound **8** (1.2900 g, 4263.7252 g/mol, 0.3026 mmol) was dissolved in anhydrous DMF in a 100 mL round-bottomed flask, then sodium azide (0.2900 g, 65.010 g/mol, 4.4609 mmol) was added. The reaction was run at 70 °C for 2 days. The mixture was filtered, rotary-evaporated, and partitioned into chloroform and water layers. The target compound was found in the organic layer. Anhydrous MgSO_4_ was added and filtered off. The filtrate was rotary-evaporated. The product was purified on a Sephadex LH-20 column with 10% methanol in chloroform. The fractions containing the target compound were combined based on the LC/MS and HPLC results and then dried before NMR analysis.

Yield = 83% (1.09 g); appearance = yellowish honey-like material. ^1^H NMR (500 MHz, acetone-*d*_6_): δ 7.83 (s, 6H), 7.32 (d, *J* = 8.1 Hz, 24H), 6.88 (d, *J* = 7.0 Hz, 24H), 4.51 (q, *J* = 5.7 Hz, 12H), 4.09 (m, 24H), 3.81 (dt, *J* = 12.1 and 4.9 Hz, 36H), 3.68–3.61 (m, 90H), 3.48 (m, 50H), 3.36 (t, *J* = 5.0 Hz, 24H). ^13^C NMR (126 MHz, acetone-*d*_6_): δ 158.08, 144.11, 131.52, 130.00, 123.68, 114.24, 78.47, 78.19, 71.55, 70.57, 70.46, 70.33, 70.20, 69.85, 69.56, 69.40, 69.33, 68.49, 67.41, 56.28, 50.51, 49.78, 47.26. HPLC: RT, 14.98 min. LC/ESI-TOF MS. Calcd exact mass for the target C_204_H_290_N_60_O_48_: 4348.2096 amu. Found: [M + 2H]^2+^ ∼ [M + 6H]^6+^; deconvoluted to 4348.2090 amu.

#### Compound **10** (G2)

Compound **9** (1.2000 g, 4,348.2096 g/mol, 0.2760 mmol) was weighed into a 35 mL CEM microwave reaction vessel. Compound **4** (1.6230 g, 372.1685 g/mol, 4.3608 mmol) was added along with copper granules (250 mg) and a stir bar. Anhydrous THF (20 mL) was introduced via a cannula needle, and the reaction mixture was purged with argon gas. The reaction was stirred continuously until all reactants had completely dissolved. The microwave reaction was run at 77 °C and 150 W for 15 h under N_2_. The reaction was completed during this period. A sticky material settled at the bottom of the vessel. LC/MS confirmed that the sticky material was the target compound. The supernatant contained the unreacted compound **4**, which was decanted. The viscous substance was subsequently dissolved in an acetone–methanol (1:1) mixture, filtered through a Celite 545 filter, and then rotary-evaporated. The product was dissolved in a mixture of methanol (10 mL) and acetone (10 mL), then treated with 10 mL of saturated EDTA solution for 1 h to remove any copper that might have formed a complex with the target compound. The mixture was rotary-evaporated and then loaded onto a Sephadex LH20 column for purification using a methanol–acetone (1:1) solution. The fractions containing the pure target compound were combined based on HPLC and LC/MS results. The product was analyzed using NMR.

Yield = 82% (2.00 g); appearance = yellowish gooey material. ^1^H NMR (500 MHz, DMSO): δ 8.01 (s, 12H), 7.91 (s, 6H), 7.80 (s, 12H), 7.21–7.16 (m, 24H), 6.81–6.76 (m, 24H), 6.57 (s, 24H), 4.50 (t, *J* = 5.2 Hz, 24H), 4.42 (d, *J* = 14.4 Hz, 36H), 3.96–3.90 (m, 24H), 3.81–3.76 (m, 48H), 3.72 (d, *J* = 7.2 Hz, 108H), 3.62–3.58 (m, 24H), 3.53 (s, 12H), 3.49 (d, *J* = 2.9 Hz, 74H), 3.33 (s, 54H), 2.28 (s, 36H). ^13^C NMR (126 MHz, DMSO): δ 157.33, 151.62, 147.83, 145.17, 143.16, 141.40, 138.61, 134.98, 133.23, 130.86, 129.70, 126.98, 126.37, 124.80, 123.83, 114.00, 106.96, 69.70, 69.53, 68.89, 68.69, 66.90, 59.00, 57.11, 55.86, 55.48, 50.16, 49.37, 49.21, 46.65, 18.68. HPLC: RT, 9.85 min. LC/ESI-TOF MS. Calcd exact mass for the target C_444_H_578_N_84_O_108_: 8,814.2319 amu. Found: [M + 3H]^3+^ ∼ [M + 12H]^12+^; deconvoluted to 8814.2607 amu.

## 3 Results

### 3.1 Chemistry

The target antioxidant dendrimers were synthesized via a divergent synthetic approach. The traditional divergent approach involves the step-by-step growth of dendrimers from the core by adding branching units until the desired size is reached. Dendrimers produced by this conventional method have multiple identical functional groups on their surfaces, and modification of some of these groups often yields inconsistent results. To prevent this, we synthesized our dendrimers by sequentially attaching two different types of building blocks—an internal building block (BB) for branching and a surface BB that imparts antioxidant properties—to the functionalized core. The surface BB was designed to contain two different types of hindered phenols, one hydrophobic and the other hydrophilic.

To synthesize the internal BB, a linker (compound **1**) was first prepared by reacting 2-[2-(2-chloroethoxy)­ethoxy]­ethanol with *p*-toluenesulfonyl (tosyl) chloride in toluene, using TEA as the base (step a, Scheme 1). After confirming the structure of compound **1** with LC/MS and NMR (spectral data are shown in Figures S1–S3), it was then conjugated to 4-hydroxybenzaldehyde to form compound **2**. While both chloro and tosyl groups of compound **1** can react with the OH group, the tosyl group reacts much faster, producing compound **2** exclusively (step b) (Figures S4–S6). It was then reacted with propargylamine in the presence of NaBH­(OAc)_3_ in THF to form the internal BB, compound **3** (step c). The purified product yielded 77%. Compound **3** was characterized by LC/MS and NMR analysis (Figures S7–S9).

**Scheme 1:**
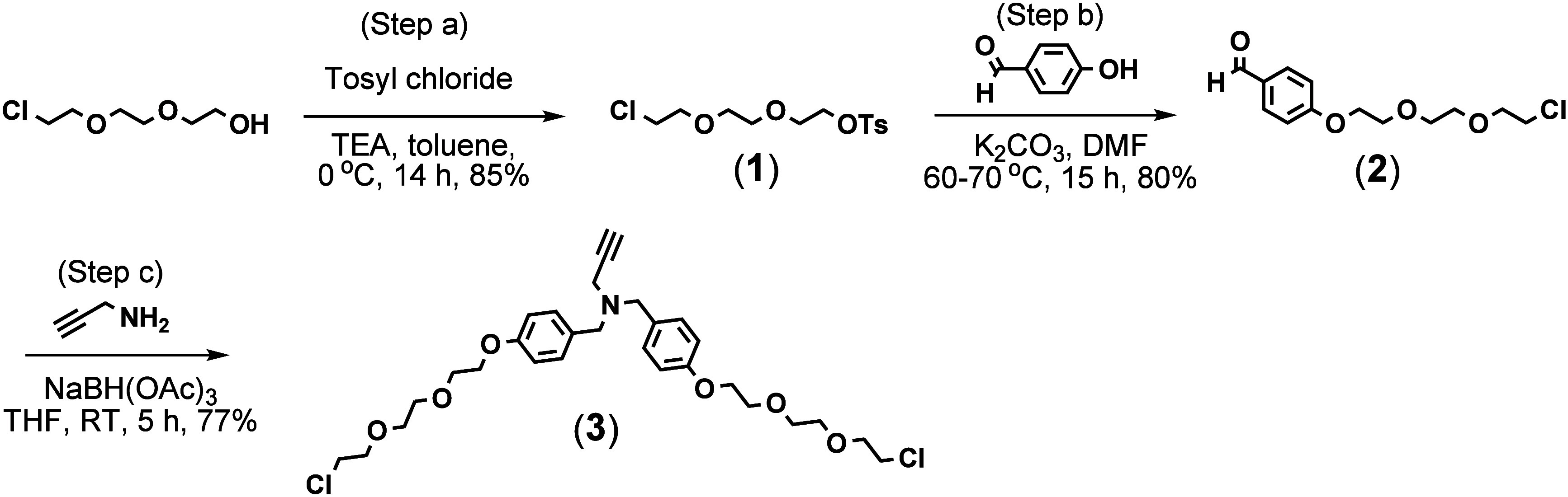
Synthesis of the Internal Building Block

The surface building block (compound **4**) is a hybrid of syringaldehyde and pyridoxal. It was synthesized via a stepwise reductive amination using 2-picoline borane in methanol (Scheme 2). Propargylamine was sequentially reacted with pyridoxal, 2-picoline borane, syringaldehyde, and then again with 2-picoline borane, which provided a product yield of 26%. When syringaldehyde was reacted with propargylamine before pyridoxal, the yield was much lower compared to when pyridoxal was added first. This difference is attributed to the difference in steric hindrance around their aldehyde groups. Pyridoxal has greater steric hindrance around its aldehyde group due to the adjacent hydroxy and hydroxymethyl substituents. In addition, pyridoxal typically remains in its cyclic hemiacetal form, formed by its aldehyde and hydroxymethyl groups. The ring must open to generate an aldehyde group before it can react with an amine. Because of these issues, it attaches slowly and gradually to propargylamine. However, it faces a significant challenge when attaching to a secondary amine that already has a syringic group attached, resulting in negligible yields. After the workup, 80% of the product was found in the organic layer (ethyl acetate) and 20% in the aqueous layer, indicating it is amphiphilic but has much higher solubility in the organic layer. The target compound was characterized with LC/MS (Figure S10) and NMR (Figures S11 and S12).

**Scheme 2:**
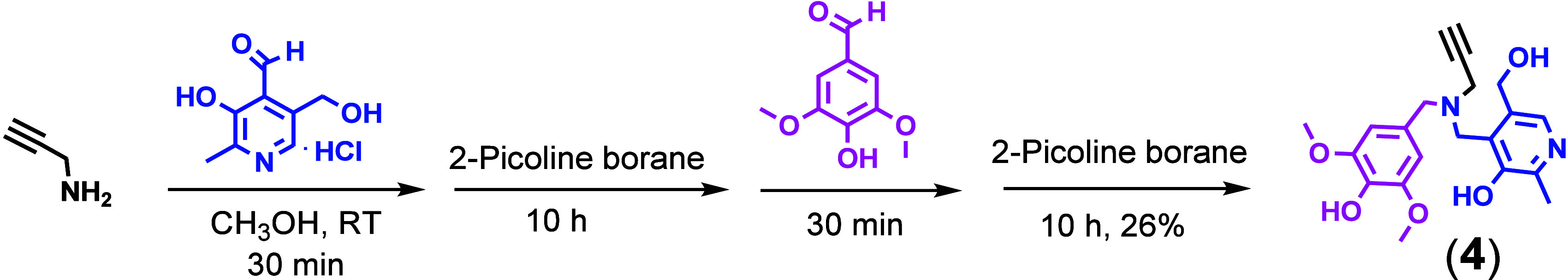
Synthesis of the Surface Building Block^a^

a Blue signifies hydrophilicity, while magenta signifies hydrophobicity.

To synthesize the target dendrimers, d-mannitol (core) was derivatized with a previously prepared linker (compound **5**) to produce compound **6** (G0.5) (step a, Scheme 3).^14^ The hydroxyl groups of d-mannitol were treated with sodium hydride (NaH) in anhydrous DMF for 1 h before adding **5**. Subsequently, the linker **5** was introduced into the reaction vessel via a cannula to minimize air ingress. This reaction involves the attachment of six linker units to the core. Although the attachment of 3–4 units occurs within 1–2 days, the reaction typically takes about a week to complete because steric congestion increases with each additional linker. Compound **6** is not UV-active; therefore, LC/MS was employed to monitor the formation of the target compound. After purification, the product was obtained in 57% yield. The purified product was analyzed with LC/MS (Figure S13). The target compound **6** appeared as [M + H]^+^ at 861.4431 amu, [M + NH_4_]^+^ at 878.4692 amu, [M + Na]^+^ at 883.4243 amu, and [M + K]^+^ at 899.3969 amu, with [M + NH_4_]^+^ being the most abundant species. The calculated masses are 861.4398, 878.4663, 883.4217, and 899.3957 amu, respectively. Additionally, compound **6** was characterized using NMR spectroscopy (Figures S14 and S15). The signal assignments were compared with the estimated NMR spectra generated by the MNova NMRPredict. In the NMR spectra of compound **6**, the chemical shifts of the first ethylene units in the six attached linkers were clearly different. However, the chemical shifts of the second ethylene units in all six chains are fairly similar to each other, indicating that the influence of electronic effects by the d-mannitol core does not extend beyond the first unit.

**Scheme 3:**
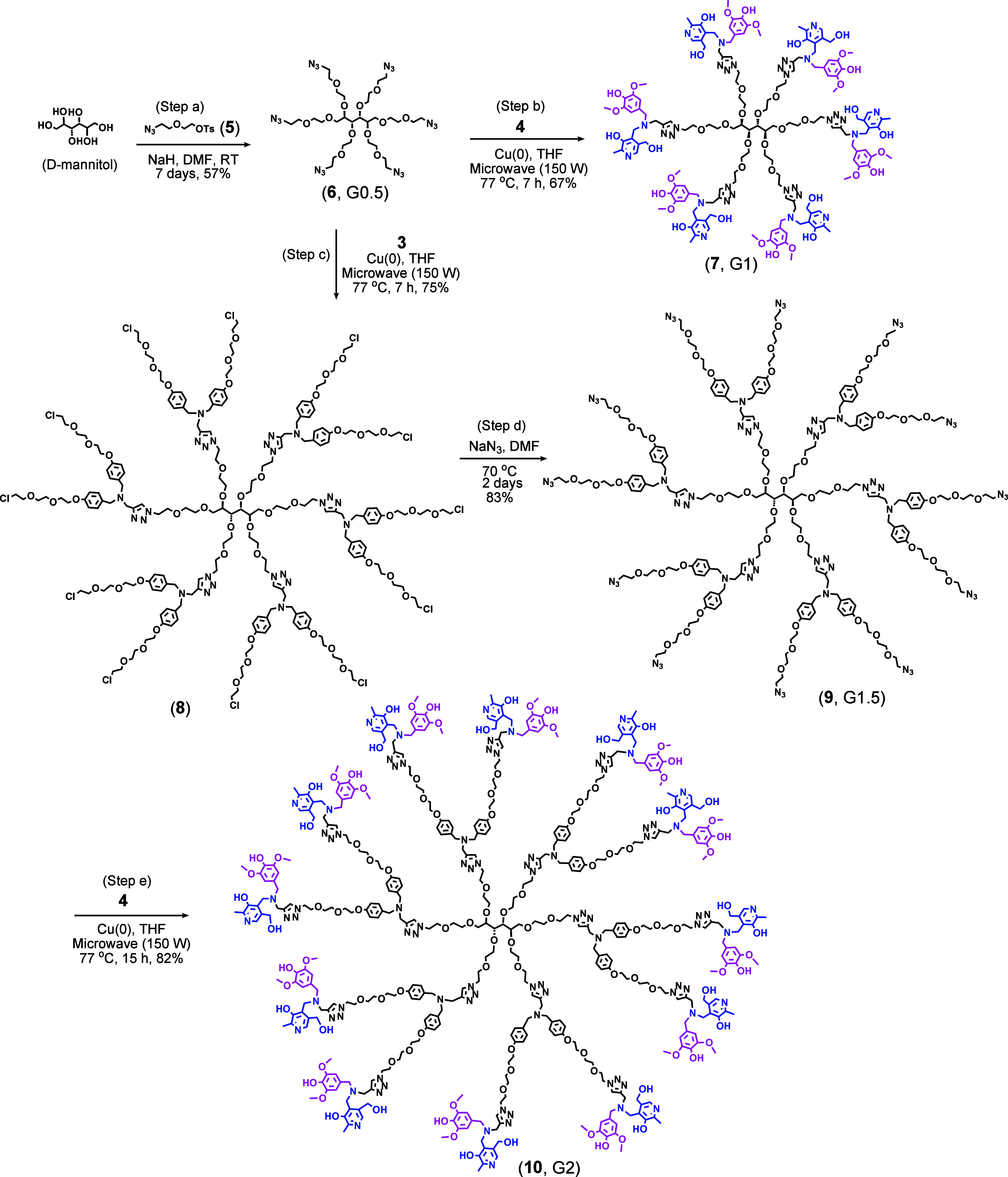
Synthesis of the Target G1 and G2 Dendrimers^a^

a Blue denotes hydrophilicity, and magenta denotes hydrophobicity.

G0.5 (compound **6**) was reacted with the surface BB (compound **4**) in anhydrous THF to synthesize G1 (compound **7**), using copper granules as the catalyst (step b). Our prior research established that copper metal granules result in markedly lower copper contamination in target antioxidant dendrimers compared to the more commonly used CuSO_4_ in alkyne–azide click chemistry.^35^ However, copper granules are much less catalytically efficient. To overcome this, all dendrimer syntheses involving copper-catalyzed alkyne–azide click reactions were conducted in a microwave at 77 °C and 150 W, greatly accelerating the process. LC/MS analysis verified complete formation of compound **7** after 7 h under these microwave conditions. Following the reaction, the residual copper granules were filtered off using a Celite 545 pad. The resulting filtrate was then treated with a saturated aqueous EDTA solution to ensure removal of any copper complexed with the target product. For purification, both silica gel column chromatography and size-exclusion chromatography were employed; however, size-exclusion chromatography on a Sephadex LH-20 column proved much more effective than silica gel at separating the target compound from small molecules, including unreacted BB and EDTA. Purification afforded the target compound **7** in 67% yield. The purified compound **7** was analyzed by LC/MS as well as NMR (Figures S16–S18). Due to its large size, it was observed in multiply charged states ranging from [M + 2H]^2+^ to [M + 6H]^6+^, which deconvoluted to 3093.4482 amu, closely matching the calculated mass of 3093.4436 amu. To assign ^1^H and ^13^C NMR signals, 2D correlation NMR techniques—including ^1^H–^1^H COSY, ^1^H–^13^C HSQC, ^1^H–^13^C HMBC, and ^1^H–^1^H NOESY—were used in conjunction with spectra predicted by an NMR estimation program.

The G0.5 was also reacted with the internal BB (compound **3**) to form compound **8** (step c), using the same methods as those used to synthesize compound **7**. After purification and characterization (Figures S19–S21), compound **8** was treated with sodium azide in DMF for 2 days, producing compound **9** (G1.5) in 83% yield (step d). Purification of G1.5 was performed via size-exclusion chromatography on a Sephadex LH-20 column, followed by LC/MS and NMR analysis (Figures S22–S24). Subsequently, G1.5 was reacted with the surface BB (**4**) in a microwave reactor at 77 °C, 150 W for 15 h to produce the G2 target compound (**10**), which contains 12 syringic units and 12 pyridoxal units (step e). As with all other microwave reactions, the reaction mixture was treated with EDTA solution and purified by size-exclusion chromatography. After purification, the reaction yield was determined to be 82%.

The purified G2 target compound was analyzed by LC/MS (Figure 1A,B) and NMR (Figure 1C,D). The compound was observed as [M + 2H]^3+^ to [M + 6H]^12+^ (Figure 1B), which were deconvoluted to 8814.2607 amu, while its calculated mass is 8814.2319 amu. In NMR analysis, the H and C signals for the key substituents were identified with the correct integrations. Since compound **10** has two different surface units, ^1^H NMR show two distinct chemical shifts for the aromatic CH groups (labeled 35 and 42 in Figure 1C), with an integration ratio of 1:2. Signals from the CH_3_ group (labeled 46), two different benzylic CH_2_ groups (labeled 33 and 39), and two OCH_3_ groups (labeled 38) also indicate that compound **10** contains two distinct aromatic rings. It should be noted that hydrogen and carbon atoms at equal distances from the core had the same chemical shifts in all six chains, except for the first ethylene units directly attached to the d-mannitol core.

**Figure 1:**
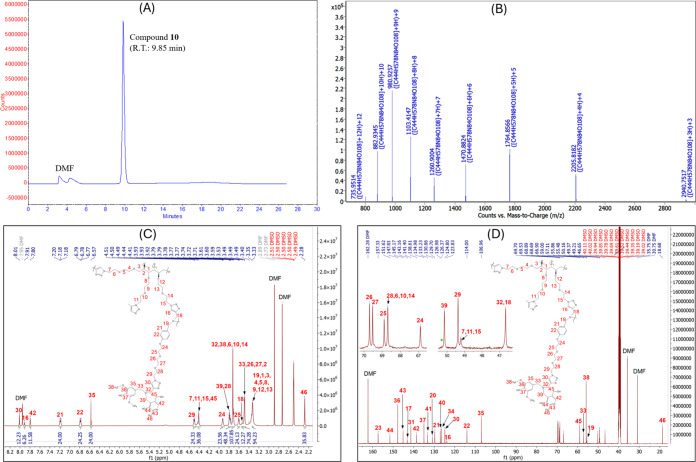
NMR analysis of compound **10** (G2): (A) HPLC; (B) MS; (C) ^1^H NMR; (D) ^13^C NMR.

### 3.2 Solubility

Antioxidants for medical applications should be sufficiently soluble in biorelevant solvents to enhance their effectiveness.

The antioxidant surface BB (**4**), consisting of one syringic unit and one pyridoxal unit, showed fairly good amphiphilicity, with 20% solubility in water and 80% in ethyl acetate during the workup. Similarly, both G1 and G2 dendrimers showed comparable amphiphilicity, although they had better solubility in organic solvents than in water. The G1 dendrimer is highly soluble in common organic solvents, including ethanol, methanol, chloroform, DMF, and DMSO. G2 is also very soluble in chloroform, DMF, and DMSO, but only sparingly soluble in methanol or acetone. However, it dissolves well in a 1:1 mixture of methanol and acetone.

Both G1 and G2 dendrimers readily dissolve in a 1:2 ethanol–water mixture at a concentration of 1 mg/mL. Furthermore, the addition of PEG600 as a cosolvent at concentrations of 1.25% and 5%, respectively, enhances their solubility in water. Overall, G1 demonstrates significantly higher water solubility than G2, requiring less cosolvent.

Similar G1 and G2 dendrimers that carry only syringic units on their surfaces showed higher hydrophobicity than the hybrid G1 (**7**) and G2 (**10**) dendrimers presented in this study. The syringaldehyde-based G1 dendrimer was soluble in methanol, acetone, chloroform, THF, DMF, and DMSO. The corresponding G2 was soluble in acetone, chloroform, DMF, and DMSO, but insoluble in methanol and ethanol. To dissolve the syringaldehyde-based G1 and G2 dendrimers in water, they required twice as much PEG600—2.5% and 10%, respectively—compared with the hybrid G1 and G2 dendrimers. A more striking difference was that the syringaldehyde-based G1 and G2 did not dissolve in the ethanol–water mixture.

This study shows that combining pyridoxal with syringaldehyde yields antioxidants with solubilities suitable for biomedical applications, representing a notable advance over other dendrimers that carry only syringic moieties.

### 3.3 Particle Size

The sizes of G1 and G2 dendrimers were measured using DLS spectroscopy. The samples were examined in various solvents, such as a mixture of ethanol and water (1:2), methanol, chloroform, and DMF. The G1 dendrimer was determined to have a diameter of 2.875 nm and a PDI of 0.1905. G2 measures 5.297 nm and has a PDI of 0.2672.

### 3.4 Radical-Scavenging Antioxidant Activity

The synthesized G1 and G2 dendrimers were evaluated for their DPPH radical-scavenging activities (Table 1). The IC50 values of the dendrimers were compared with those of the starting materials, syringaldehyde and pyridoxal.

**Table 1:** IC50 Values of Antioxidants Measured in the DPPH Assay

antioxidant	IC50 (μM)	antioxidant	IC50 (μM)
compound **10** (G2)	1.28	syringaldehyde	200
compound **7** (G1)	2.6	pyridoxal	16500
compound **4** (BB)	15.2		

The IC50 values for G2 and G1 are 1.28 and 2.6 μM, respectively. In comparison, the IC50 value for the surface BB (**4**) is 15.2 μM, whereas those for syringaldehyde and pyridoxal are 200 and 16500 μM, respectively.

Based on these IC50 values, G2, which contains 12 syringic units and 12 pyridoxal units, is twice as effective at DPPH radical scavenging as G1, which has 6 syringic units and 6 pyridoxal units. The DPPH radical-scavenging activity of G2 is 156 times greater than that of syringaldehyde and 12890 times more potent than pyridoxal. Similarly, G1 is 77 times more effective than syringaldehyde and 6346 times more potent than pyridoxal in DPPH radical scavenging. Surprisingly, the surface BB, which consists of only one of each phenolic unit, shows fairly good radical-scavenging activity, being 13 times more effective than syringaldehyde and 1085 times more effective than pyridoxal.

The radical-scavenging activities of G2 and G1 dendrimers were compared to those of monomeric antioxidants, with normalization based on the number of phenolic groups present on each dendrimer’s surface. Because the dendrimer interiors do not participate in radical scavenging, normalizing dendrimer concentrations by the number of surface phenolic units yields an inaccurate comparison. Additionally, these dendrimers contain two types of phenolic units (syringic and pyridoxal). A better normalization is to divide the IC50 values of monomeric antioxidants by 24 for comparison with G2 and by 12 for G1. This adjustment makes G2 6.5 times more effective than syringaldehyde and 537 times more effective than pyridoxal. Likewise, G1 exceeds syringaldehyde by 6.4 times and pyridoxal by 529 times. Even after normalization, our G2 and G1 dendrimers remain significantly better antioxidants than their monomeric counterparts, highlighting the benefits of organizing small antioxidants within a dendritic framework.

### 3.5 Cell Viability and Anti-Inflammatory Activity

The dendrimers were tested for cytotoxicity in RAW 264.7 macrophages and then assessed for anti-inflammatory activity in LPS-stimulated macrophages. Macrophages are the first line of defense against infection and promote an inflammatory response by releasing mediators, such as NO and IL-6. NO is associated with antimicrobial activity in the local environment and triggers the production of proinflammatory cytokines to mount a more widespread immune response. IL-6 amplifies the inflammatory response and is associated with several chronic inflammatory conditions.

Cell viability was assessed after 24 h of exposure to G1 (compound **7**) at concentrations ranging from 0 to 323 μM and G2 (compound **10**) at concentrations from 0 to 113 μM in RAW 264.7 macrophage cells. A decrease in cell viability caused by G1 was observed at a concentration of 323 μM (Figure 2A). Conversely, G2 caused a statistically significant reduction in cell viability at a much lower concentration, starting at 11.3 μM (Figure 2B). The LD50 values for G1 and G2 were found to be 129 and 32 μM, respectively.

**Figure 2:**
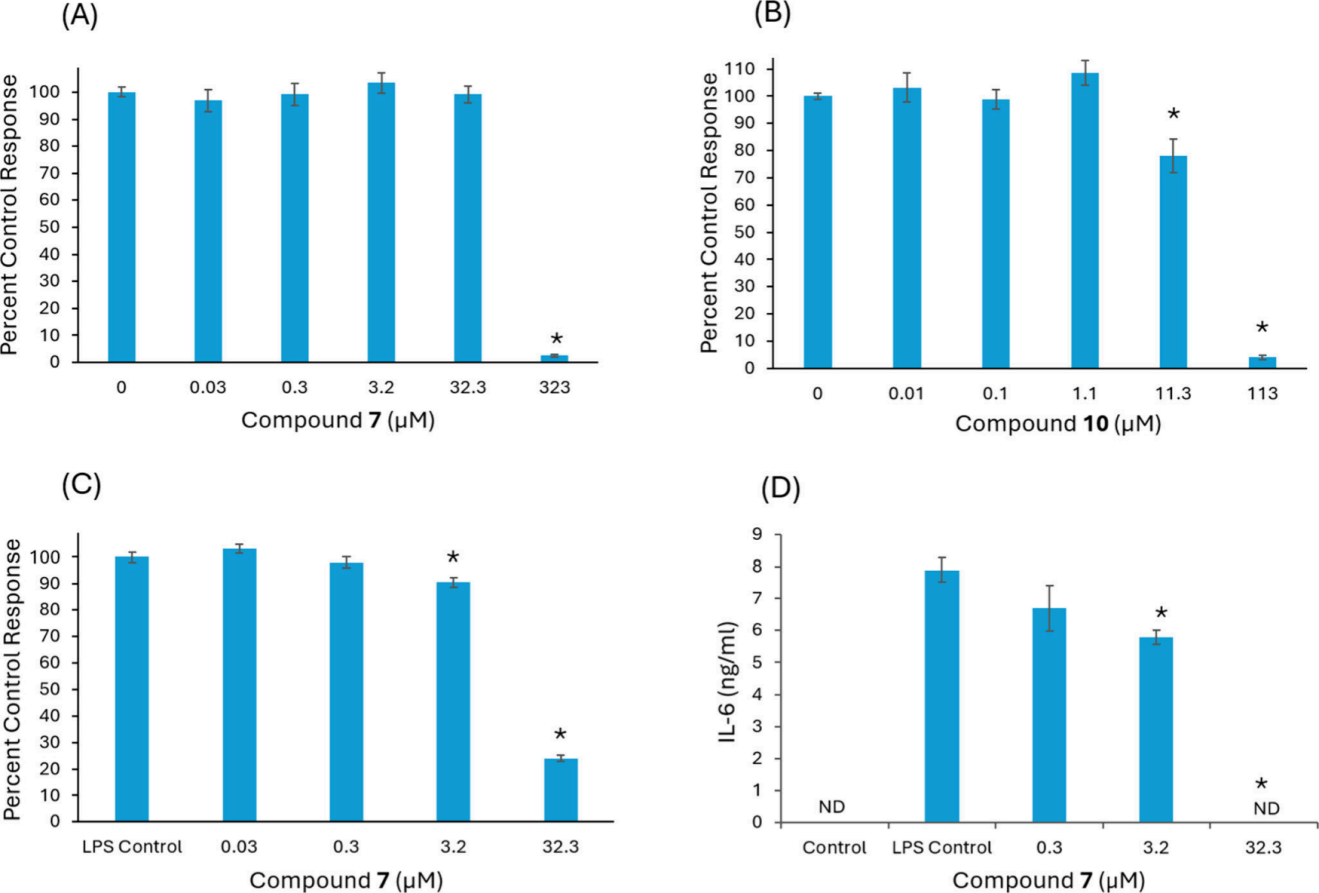
Cell viability and anti-inflammatory activity in LPS-stimulated RAW 264.7 macrophages following exposure to compounds **7** (G1) and **10** (G2): (A) cell viability of G1; (B) cell viability of G2; (C) effects of G1 on NO scavenging; (D) effects of G1 on reducing IL-6 levels. The asterisk (*) indicates a significant difference from the untreated control (*p* < 0.05).

The anti-inflammatory activities of G1 and G2 were assessed by measuring their effects on NO and IL-6 levels in an LPS-stimulated model following a 6-h pretreatment with nontoxic concentrations of G1 (0–32.3 μM) and G2 (0–1.1 μM). NO levels were quantified as a percent control response, with the LPS control group set at 100% to indicate maximal NO production. G1 at concentrations of 0.03 and 0.3 μM did not significantly alter NO levels (Figure 2C). However, a statistically significant reduction in NO levels was observed at 3.2 μM, where the percent control response decreased to approximately 90.7%, indicating that 9.3% of NO was scavenged. A more pronounced effect was seen at 32.3 μM, where the response dropped to approximately 24%, corresponding to a 76% reduction in NO levels. It is important to note that macrophages produce NO constitutively, regardless of LPS presence, which may result in detectable NO even at the highest concentration.

IL-6 levels were measured in ng/mL and converted to percentage reduction relative to the LPS control. Treatment with G1 at 3.2 μM caused a 27% reduction in IL-6 levels, while 32.3 μM resulted in a complete 100% reduction (Figure 2D). These findings indicate that G1 significantly suppresses IL-6 production in a dose-dependent manner.

Together, these results demonstrate that G1 exerts dose-dependent anti-inflammatory effects, with significant reductions in both NO and IL-6 levels observed at concentrations ≥ 3.2 μM. In comparison, G2 had no significant impact on these inflammatory mediators at its nontoxic test concentrations (0–1.1 μM) (data not shown). Overall, the cell-based results indicate that the G1 dendrimer exhibits significantly higher cell viability and superior anti-inflammatory activity than the G2 dendrimer.

## 4 Discussion

One of the primary advantages of developing macromolecular antioxidants in dendritic structures is that they enable multifunctionality on a single platform. Compared with other types of nanoantioxidants, their surface and internal-cavity chemistries, as well as the degree of branching, can be more readily modified to achieve multifunctionality at appropriate sizes within well-defined structures. Although antioxidant potency can be enhanced by various other methods, building antioxidants into dendritic architectures helps overcome common limitations associated with natural antioxidants and simultaneously improves their radical-scavenging efficiency. The present study aimed to enhance the solubility of antioxidant dendrimers by employing surface-building blocks containing two distinct hindered phenols with varying solubilities, while maintaining effective radical-scavenging and anti-inflammatory properties.

The results of this research reveal several key findings. Primarily, the G1 and G2 dendrimers, which contain 6 and 12 sets of syringic and pyridoxal units, respectively, exhibit notable DPPH radical-scavenging activity (IC50 of G1 = 2.6 μM and G2 = 1.28 μM). Given that syringaldehyde (200 μM) and pyridoxal (16500 μM) alone show minimal DPPH radical-scavenging effects, the radical-scavenging activities of the dendrimers are remarkable. Moreover, the surface BB, which contains only one of each unit, showed a dramatically higher IC50 value (15.2 μM) compared to its monounit counterparts, clearly indicating that the functional groups on the phenolic units play a very important role in radical scavenging. By attaching syringaldehyde and pyridoxal to the amino group via reductive amination, their electron-withdrawing aldehyde groups are transformed into electron-donating benzylic groups. This indicates that phenolic aldehydes, particularly hindered phenolic aldehydes containing *o*-EDGs relative to the OH group, can form antioxidants with significantly higher activity than the aldehydes alone when attached to other scaffolds. Another study reported similar findings, noting that syringaldehyde exhibits strong antioxidant effects when conjugated to L-tryptophan.^36^ Pyridoxal is a member of the vitamin B6 family and is water-soluble. A computational study reported that pyridoxal exhibits effective free-radical-scavenging, particularly for hydroxyl (HO^•^) and nitrogen dioxide (NO_2_^•^) radicals in aqueous environments.^37^ Although its efficient radical-scavenging effects against HO^•^ and NO_2_^•^ cannot be directly extrapolated to DPPH radicals, pyridoxal alone did not exhibit strong radical-scavenging activity against DPPH radicals in our study.

Our results indicate that the hydrophobicity of dendrimers can be reduced without compromising their radical-scavenging activity by attaching water-soluble molecules alongside hydrophobic antioxidant units to their surfaces. Although previous studies have shown that the hydrophobicity of hindered phenols, such as syringaldehyde, can be overcome when they are conjugated to a water-soluble scaffold,^38^ developing water-soluble antioxidant dendrimers that carry multiple hindered phenolic units remains challenging because hindered phenols are inherently hydrophobic, and their hydrophobicity can increase with the number of units. As shown in our previous studies, hindered phenolic units exhibit strong radical-scavenging activity without inducing pro-oxidant activity when incorporated into dendritic frameworks.^13,14^ To maximize the benefits of hindered-phenol-based antioxidant dendrimers in biomedical applications, it is essential to improve their water solubility. The research findings indicate that fully water-soluble antioxidant dendrimers may be synthesized by alternately attaching hindered phenols and highly water-soluble molecules to their surfaces.

The anti-inflammatory effects of dendrimers were evaluated by measuring their impact on levels of key inflammatory mediators, NO and IL-6, in an LPS-stimulated macrophage model. NO is typically produced early in the inflammatory response and acts locally, often targeting nearby bacterial cells. In contrast, IL-6 acts on other cells and exerts its effects over a longer duration by activating T cells and initiating a comprehensive immune response. Both markers have also been associated with chronic inflammation. Both are activated by similar pathways: MAP Kinase and NFkB (i.e., LPS-MAPK-NFkB-iNOS-NO and LPS-MAPK-NFkB-IL6), which are the general inflammatory pathways activated by LPS. However, which MAPKs are activated can vary and depend on the cell line. In addition, LPS can activate ROS-MAPK-NFkB-IL-6. This indicates that the activation pathways are similar and may overlap, but they can also have different upstream mediators, suggesting that NO and IL-6 are not directly dependent on one another. The modulation of NO and IL-6 is indicative of therapeutic potential for inflammatory conditions.

Our findings indicate that G1 exhibits dose-dependent inhibitory effects on NO and IL-6 levels. At concentrations ≤0.3 μM, G1 has a limited impact on NO levels; however, concentrations ≥3.2 μM lead to significant reductions, highlighting its role as a NO scavenger that may help mitigate oxidative stress and inflammation. Regarding IL-6, treatment with G1 leads to marked suppression of IL-6 levels in a dose-dependent manner. Specifically, a concentration of 3.2 μM results in a 27% reduction, while 32.3 μM achieves a complete 100% reduction. This strong inhibitory effect underscores G1’s potential in cytokine modulation. The mechanisms for IL-6 level reduction are multifaceted, involving direct interaction with IL-6, interference with its receptor binding, inhibition of IL-6 signaling pathways, suppression of upstream activators for IL-6 production, and downregulation of IL-6 gene expression.^39^ Although our preliminary study does not pinpoint the exact mechanism by which G1 reduces IL-6 levels, the observed suppression of IL-6 is particularly significant given IL-6’s role in promoting inflammatory signaling and its association with chronic inflammatory diseases. Taken together, these results clearly indicate that G1 has a dual capacity to reduce both NO and IL-6 levels, thereby exerting anti-inflammatory effects by scavenging reactive radical species while simultaneously inhibiting pro-inflammatory cytokine production. The dose-dependent nature of these effects underscores the importance of optimizing concentration to achieve therapeutic efficacy. Future research should further investigate the molecular pathways involved in G1′s action and evaluate its *in vivo* efficacy and safety profile.

To our surprise, the G2 dendrimer shows minimal effect on reducing inflammatory markers. Although G2 showed good DPPH radical-scavenging effects (IC50 of 1.28 μM), these effects were not extrapolated to anti-inflammatory actions on macrophages. One possible reason could be the test concentrations (0–1.1 μM) of G2 that we used to stay within its nontoxic range. The concentrations might have been too low to detect measurable effects. Its size might be another reason that has affected the results. Since the NO assay only measures extracellular NO, our results might not reflect its cellular uptake efficiency related to size. However, it is reasonable to think that the large G2 dendrimer is less effective at neutralizing rapidly diffusing NO. Previous studies mention that small-sized antioxidants are preferred in biological settings because they tend to have higher diffusibility and transmembrane permeability, contributing to high cellular uptake efficiency.^40–42^ However, they are less stable and less potent in *in vivo* systems.^40,43,44^ Large antioxidants with limited diffusion are useful for applications, such as drug delivery or targeted delivery to specific tissues or organs.^45,46,30^ On this basis, we can infer that intermediate-sized antioxidants, which fall between small and large antioxidants, could offer a good balance of diffusibility, solubility, reactivity, stability, and bioavailability. The G1 might belong to this intermediate-sized category, producing more effective anti-inflammatory activities than the larger G2. Nevertheless, it is premature to draw definitive conclusions from this preliminary study. It remains possible that the effects of size on anti-inflammatory activity, as measured by various inflammatory markers, may differ, as documented in a prior study of G4–G6 PAMAM dendrimers terminated with hydroxyl groups (specifically, aminoethyl ethanolamine [AEEA]). This study reported generation-dependent effects on COX-2 enzyme inhibition that increased with higher dendrimer generations.^47^

The typical particle size range used in nanomedicine is 1–100 nm (ISO and scientific consensus).^48^ Particles at this scale are reported to effectively interact with biological systems, such as penetrating cells, crossing biological barriers, and circulating in the bloodstream.^49–52^ However, in practice, some nanomedicine particles can be as large as 300 nm or even extend up to 1000 nm (1 μm), especially when designed for controlled drug release or targeting specific organs.^53^ Nanoparticles, including those functionalized as nanoantioxidants, can be internalized by cells through endocytosis or phagocytosis.^50,54,55^ Phagocytic cells (e.g., macrophages) can engulf large particles (>500 nm) through phagocytosis.^56–58^ Nonphagocytic cells (e.g., epithelial or endothelial cells) typically rely on endocytosis, which preferentially takes up small particles (often <100 nm).^57,59,60^ According to DLS data, our G1 and G2 dendrimers measure 2.875 and 5.297 nm, respectively. This indicates that both G1 and G2 are well within the size range that can be taken up by cells, including macrophages. In fact, a previous study has shown that a neutral G4 PAMAM dendrimer terminated with OH (PAMAM-OH), similar in size (∼4 nm) to our G2, is taken up by brain macrophages activated in the setting of neuroinflammation.^61^ The study reports that the neutral G4 PAMAM-OH dendrimer, conjugated to a few FITC dye molecules and without targeting ligands, exhibits significant uptake by activated microglia and astrocytes in neuroinflammatory regions, whereas free FITC exhibits a nonspecific distribution. Cells not participating in inflammatory processes do not substantially take up the conjugate, indicating that the targeted uptake is associated with intrinsic properties of the dendrimer. Moreover, uptake of the dendrimer conjugate in healthy kits is minimal, whereas cerebral palsy kits show much higher uptake in activated microglia and astrocytes, suggesting that this increased uptake is associated with increased phagocytic activity and scavenger receptor expression during inflammation. Given these findings, it is unlikely that the limited anti-inflammatory activity of our G2 dendrimer can be attributed to its size. A notable difference between PAMAM dendrimers and our G2 dendrimer is the presence of hydrophobic phenolic units on the surface of our G2 dendrimer, which can decrease its solubility in cytosolic media and thereby modulate cellular uptake, membrane interactions, and ultimately anti-inflammatory activity. Further studies on the subcellular localization of both G1 and G2 dendrimers will help gain deeper insights into their mechanisms of action and the optimal size for maximal anti-inflammatory effects without compromising cell viability. Another study involving various G4 PAMAM dendrimers terminated with NH_2_, OH, or COOH showed that these dendrimers independently reduced NO levels in LPS-stimulated rat peritoneal macrophages, with NH_2_ > OH > COOH, suggesting that surface groups on dendrimers are important for anti-inflammatory potency.^47^ Additionally, the study reports that dendrimers with different surface groups exhibit varying inhibitory effects on COX enzymes: the G4 PAMAM-NH_2_ dendrimer inhibits both COX-1 and COX-2, with a stronger effect on COX-2. G4 PAMAM-OH (AEEA) shows comparable inhibition of both COX-1 and COX-2, but certain OH-terminated dendrimers selectively target COX-2. Furthermore, G4 PAMAM-NH_2_ and its indomethacin complex showed higher anti-inflammatory activity than free indomethacin in an adjuvant-induced arthritis assay. In terms of radical-scavenging ability, our dendrimers outperform G4 PAMAM-NH_2_ dendrimers (data not shown), yet our G2 dendrimer still lacks anti-inflammatory properties. Building on this earlier study, encapsulating or attaching anti-inflammatory drugs to our dendrimers may improve their efficacy by scavenging radicals and inhibiting molecular pathways involved in inflammation.

According to the literature, the anti-inflammatory effect is mediated by multiple biochemical pathways and may differ among cell types. We are currently testing these antioxidant dendrimers in various cell lines to assess their therapeutic potential for the management of inflammatory conditions.

## 5 Summary and Conclusion

This research employed an innovative method to develop effective macromolecular dendritic antioxidants by attaching two distinct hindered phenolic units with different solubilities to their surfaces. The G1 dendrimer contains six sets of hydrophobic syringaldehyde and hydrophilic pyridoxal units arranged alternately, while G2 contains 12 sets of these units. Both G1 and G2 dendrimers, composed of multiple hindered phenolic units, were found to be amphiphilic and exhibited significantly improved water solubility compared to dendrimers containing only syringaldehyde units.

In the DPPH assay, G2 was twice as effective as G1 at scavenging DPPH radicals, with activity 156 times higher than syringaldehyde and 12890 times more potent than pyridoxal. G1 was 77 times more effective than syringaldehyde and 6346 times more effective than pyridoxal, demonstrating the benefits of antioxidants in dendritic structures. In terms of cytotoxicity toward macrophages, G1 had a much more favorable toxicity profile than G2: G1 caused a significant reduction in cell viability only at 323 μM, whereas G2 reduced cell viability at concentrations as low as 11.3 μM. G1 exhibited notable anti-inflammatory activity by reducing NO and IL-6 levels in LPS-stimulated macrophages in a dose-dependent manner. Specifically, G1 showed a statistically significant reduction in NO levels (9.3%) at 3.2 μM, with a more pronounced NO scavenging effect at 32.3 μM (76%). G1 caused a 27% decrease in IL-6 levels at 3.2 μM and a complete 100% reduction at 32.3 μM. These results suggest that G1 exerts its strong anti-inflammatory effects through a dual mechanism: scavenging ROS and reducing pro-inflammatory cytokine production. In comparison, G2 had no significant effect on these inflammatory mediators at its nontoxic test concentrations (0–1.1 μM).

Overall, the innovative methods used in this study will lay the foundation for future development of dendritic antioxidants, potentially improving therapeutic outcomes by fighting inflammation in antioxidant therapy through multiple mechanisms: scavenging free-radical species and suppressing pro-inflammatory cytokine expression.

## Supplementary Material



## Data Availability

The original contributions presented in this study are included in the article or Supporting Information. Further inquiries can be directed to the corresponding author.

## Notes

Characterization and supporting figures including MS and ^1^H and ^13^C NMR spectra (PDF)
