# Supplementary material for: Amphiphilic Macromolecular Dendritic Antioxidants with Surfaces Coated in Hybrid Phenolic Units That Provide Anti-Inflammatory Properties
Source: ACS Appl Polym Mater. 2026 Feb 20;8(5):3445–58. doi: 10.1021/acsapm.5c04280 (PMC12993807; doi:10.1021/acsapm.5c04280)

Supporting Information

Amphiphilic Macromolecular Dendritic Antioxidants with Surfaces Coated in Hybrid Phenolic Units That Provide Anti-Inflammatory Properties

Blessed Agbemade,[1,2] Fati Haruna,[1] Aundrea Stengard,[1] Nanzhu Li,[1] Cal M. Butts,[1] Rebecca Uzarski,[3] and Choon Young Lee*,[1,2]

1. Department of Chemistry and Biochemistry, Central Michigan University, Mount Pleasant, MI 48859, USA
2. Science of Advanced Materials Program, Central Michigan University, Mount Pleasant, MI 48859, USA
3. Department of Biology, Central Michigan University, Mount Pleasant, MI 48859, USA

Corresponding author: Choon Young Lee (Professor). Contact email: lee1cy@cmich.edu. Contributing author emails: agbem1b@cmich.edu (B.A.), harun1f@cmich.edu (F.H.), steng1ae@cmich.edu (A.S.), li3n@cmich.edu, butts1cm@cmich.edu (C.B.), uzars2rl@cmich.edu (R.U.)

Table of Contents

1. **Characterization**--3

2. **Supporting Figures**

S1. MS Spectrum of Compound **1** --4
S2. ^{1}H NMR Spectrum ($CDCl_3$, 32 scans) of Compound **1**-------------------------5
S3. ^{13}C NMR Spectrum ($CDCl_3$, 1024 scans) of Compound **1**---------------------6
S4. MS Spectrum of Compound **2**---7
S5. ^{1}H NMR Spectrum ($CDCl_3$, 32 scans) of Compound **2**-------------------------8
S6. ^{13}C NMR Spectrum ($CDCl_3$, 1024 scans) of Compound **2**---------------------9
S7. MS Spectrum of Compound **3**--10
S8. ^{1}H NMR Spectrum ($CDCl_3$, 32 scans) of Compound **3**------------------------11
S9. ^{13}C NMR Spectrum ($CDCl_3$, 1024 scans) of Compound **3**--------------------12
S10. MS Spectrum of Compound **4**---13
S11. ^{1}H NMR Spectrum ($CDCl_3$, 32 scans) of Compound **4**----------------------14
S12. ^{13}C NMR Spectrum ($CDCl_3$, 1024 scans) of Compound **4**------------------15
S13. MS Spectrum of Compound **6**---16
S14. ^{1}H NMR Spectrum (D-Acetone, 32 scans) of Compound **6**-----------------17
S15. ^{13}C NMR Spectrum (D-Acetone, 1024 scans) of Compound **6**-------------18
S16. MS Spectrum of Compound **7**---19
S17. ^{1}H NMR Spectrum (MeOD, 32 scans) of Compound **7**----------------------20
S18. ^{13}C NMR Spectrum (MeOD, 1024 scans) of Compound **7**------------------21
S19. MS Spectrum of Compound **8**---22
S20. ^{1}H NMR Spectrum ($CDCl_3$, 32 scans) of Compound **8**----------------------23
S21. ^{13}C NMR Spectrum ($CDCl_3$, 1024 scans) of Compound **8**------------------24
S22. MS Spectrum of Compound **9**---25
S23. ^{1}H NMR Spectrum (D-Acetone, 32 scans) of Compound **9**-----------------26
S24. ^{13}C NMR Spectrum (D-Acetone, 1024 scans) of Compound **9**-------------27

1. Characterization

^{1}H NMR spectra were recorded on a 500 MHz NMR spectrometer (Bruker, USA). The samples were prepared in $CDCl_3$, D-acetone, or D-methanol (MeOD) at a concentration of 20 mg/mL. The NMR solvent used for each compound was specified in both the NMR spectra and the corresponding synthesis section. ^{13}C NMR spectra were recorded on a 125 MHz NMR spectrometer (Bruker, USA). The NMR data were processed using the MNova NMR program (version 16.0). The MNova NMRPredict program (version 16.0) was used to help assign the ^{1}H and ^{13}C NMR signals.

For each NMR spectrum, a detailed compound structure with all H and C atoms numbered is included to facilitate NMR signal assignment. However, for larger molecules—from Generation 0.5 (compound **6**) to Generation 2 (compound **10**, as shown in the main text)—not all atoms were numbered because the D-mannitol-based dendrimers showed apparent symmetry in their chemical shifts. It is important to note that unnumbered atoms have the same chemical shifts as similar functional groups positioned at equal distances from the D-mannitol core. The numbered atoms represent functional groups throughout the entire molecule.

Mass spectra were obtained using either an ultra-performance liquid chromatography (UPLC)-electrospray ionization (ESI)-quadrupole time-of-flight (Q-TOF) mass spectrometer (AdvanceBio 6545XT, Agilent, USA) or a high-performance liquid chromatography (HPLC)-electrospray ionization (ESI)-time-of-flight (TOF) mass spectrometer (G6230B, Agilent, USA). Both the UPLC and HPLC systems are equipped with a multi-wavelength diode array detector (Agilent, USA).

The LC/MS samples were prepared at a concentration of 1 ng/mL. For fractions obtained from column chromatography, samples were prepared by performing three serial dilutions in the matrix (water-acetonitrile, 50:50, with 0.1% formic acid). Prior to injection, all samples were filtered through a syringe filter (Minisart RC4, cellulose membrane, 0.2 μm pore size; Sartorius, UK). The injection volume was 0.3 μL. LC separations were conducted on an Agilent C18 column (InfinityLab Poroshell 120 EC-C18 with a mean particle size of 1.9 μm, 2.1 mm inner diameter, and 50 mm length) utilizing a water-acetonitrile gradient system (from 5% to 95% acetonitrile) containing 0.1% formic acid. The flow rate was 0.4 mL/min for 10 min, with the column oven temperature kept at 35 °C. Mass analysis of all samples was performed using the Dual Agilent Jet Stream Electrospray Ionization (AJS ESI) as the ion source under these conditions.

2. Supporting Figures

S1. MS Spectrum of Compound **1**.

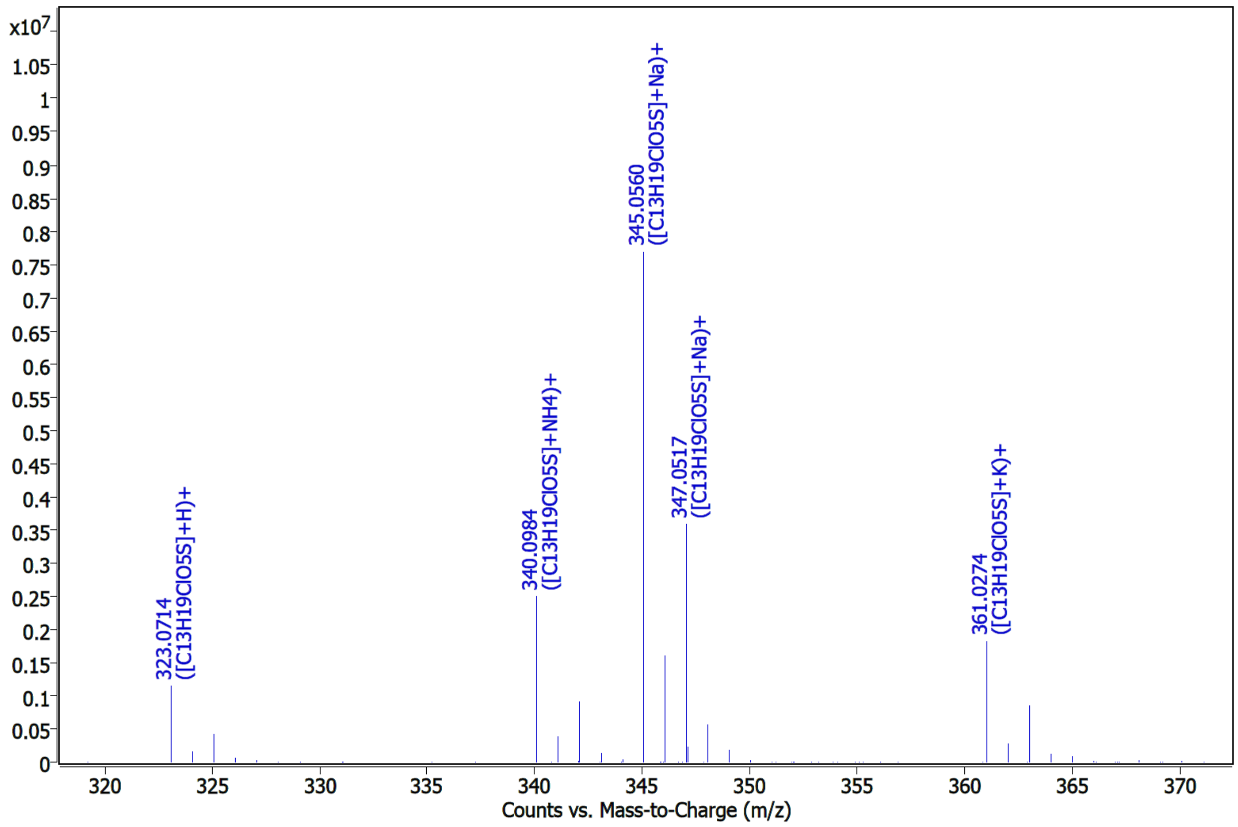

S2. ^{1}H NMR Spectrum ($CDCl_3$, 32 scans) of Compound **1**.

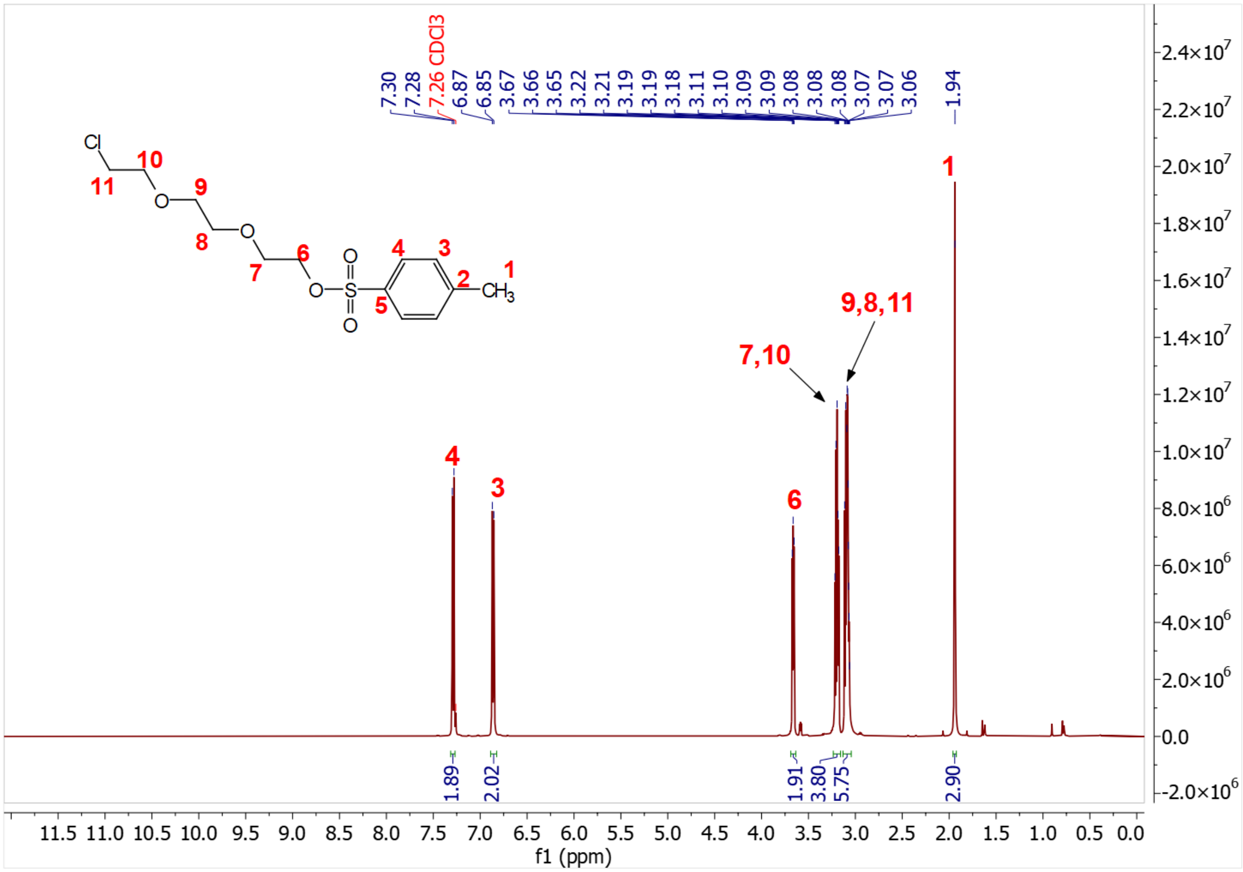

S3. ^{13}C NMR Spectrum ($CDCl_3$, 1024 scans) of Compound **1**.

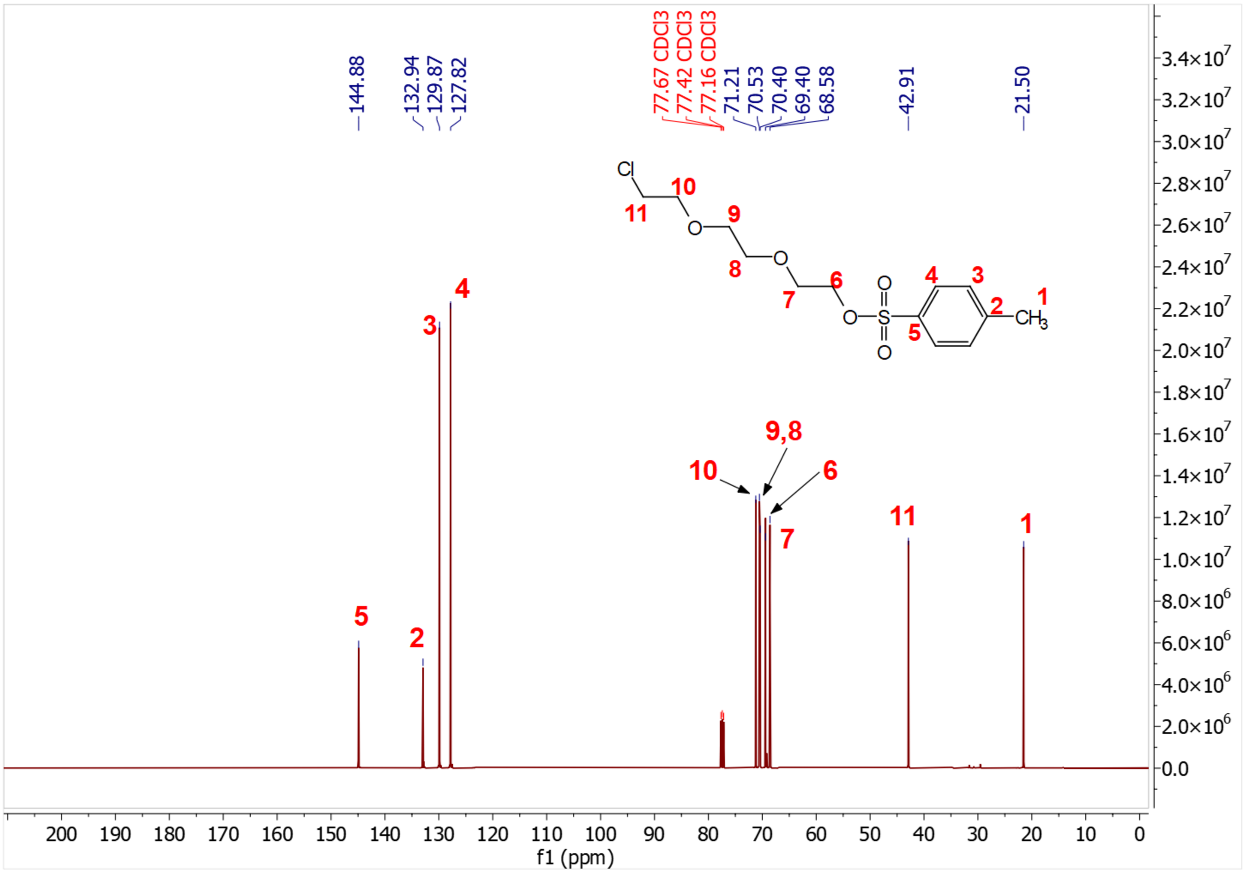

S4. MS Spectrum of Compound **2**.

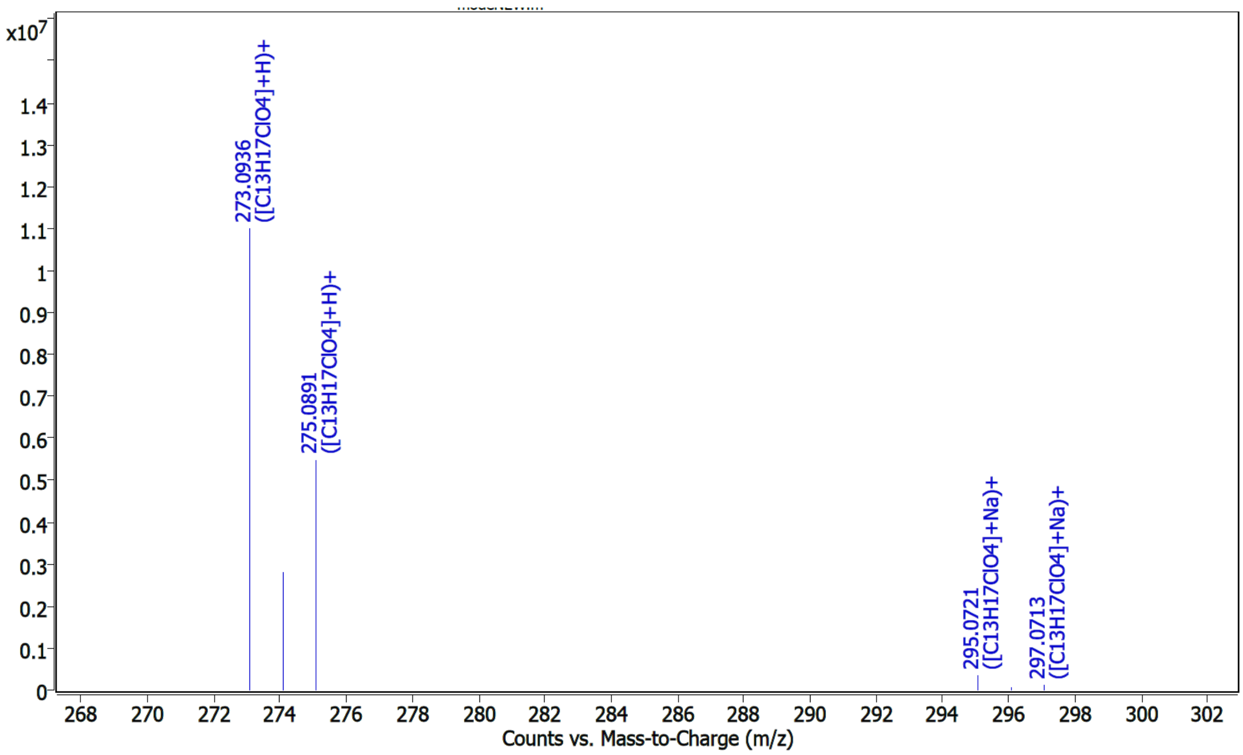

S5. ^{1}H NMR Spectrum ($CDCl_3$, 32 scans) of Compound **2**.

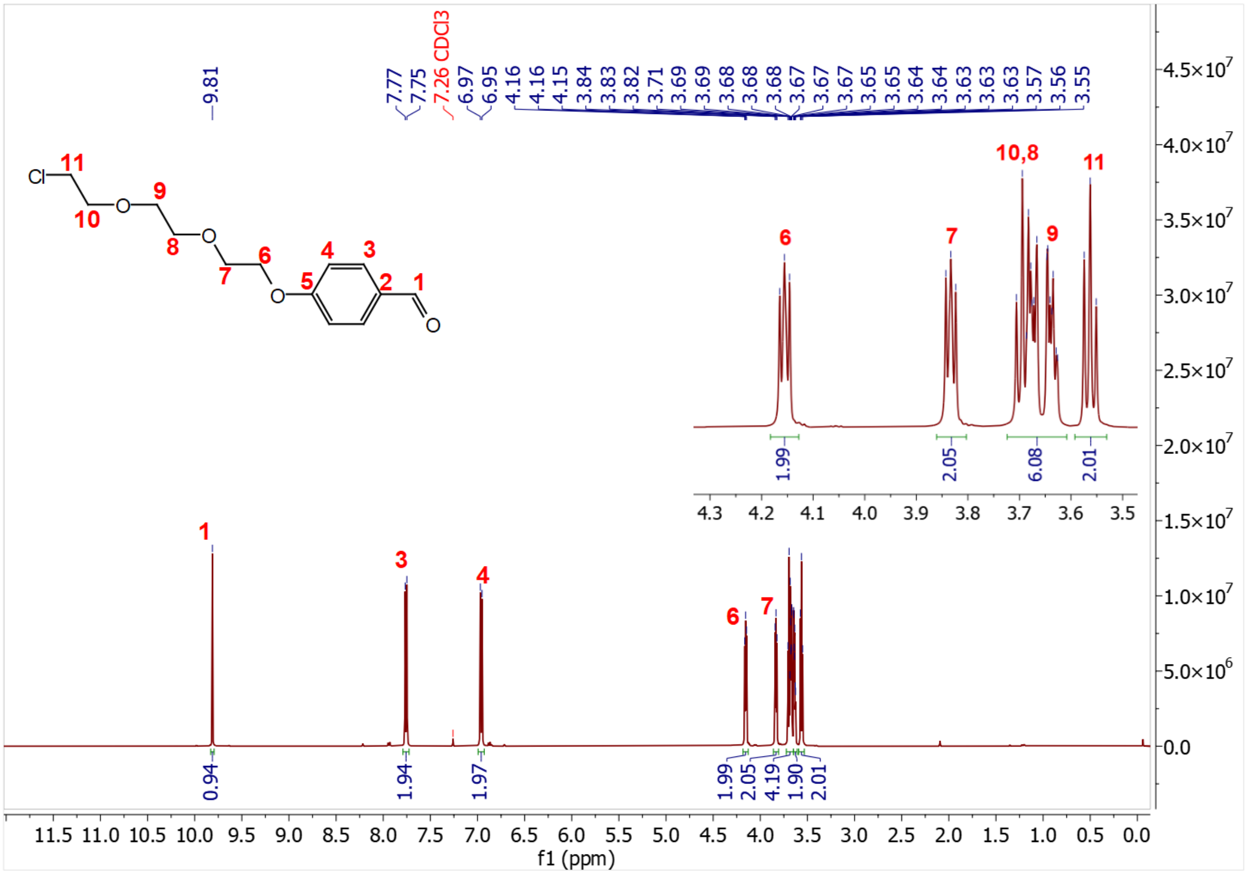

S6. ^{13}C NMR Spectrum ($CDCl_3$, 1024 scans) of Compound **2**.

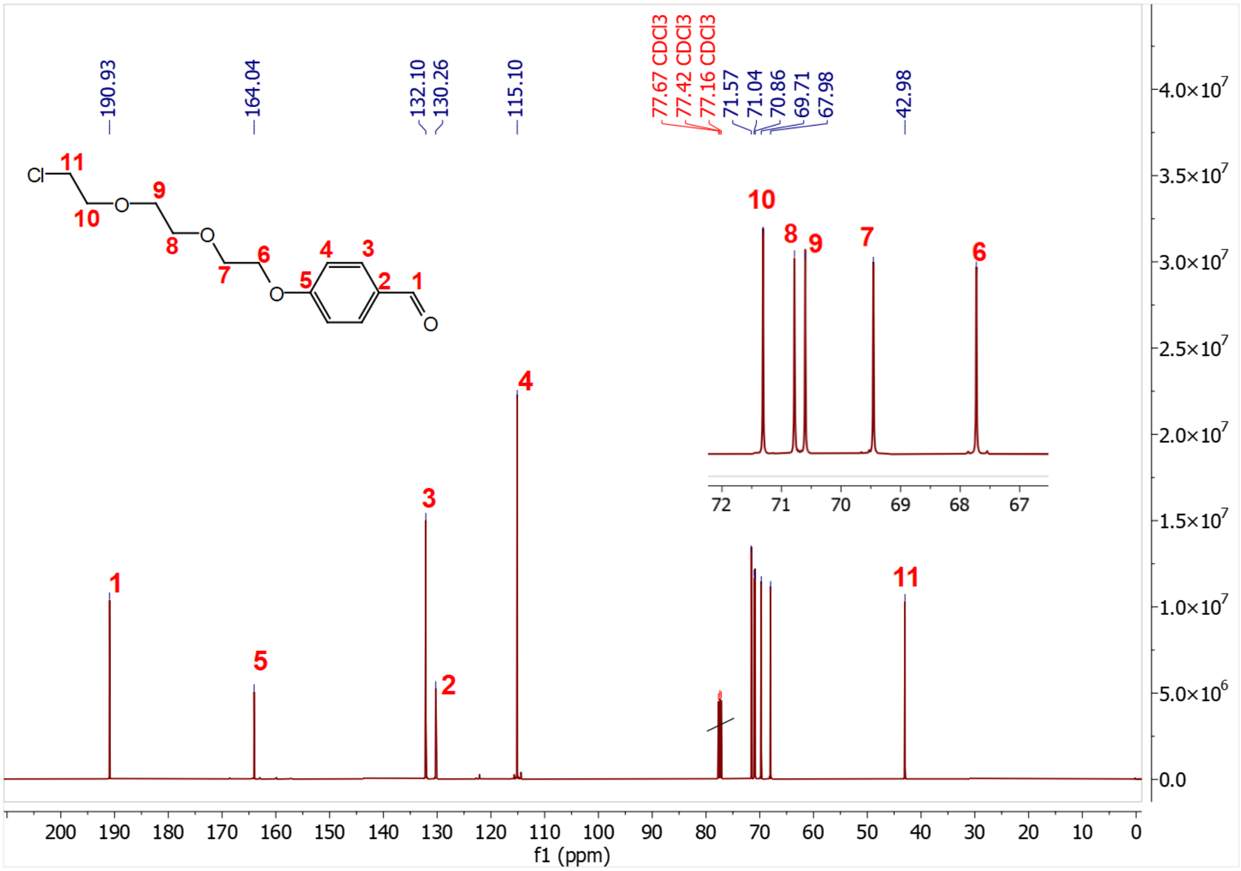

S7. MS Spectrum of Compound **3**.

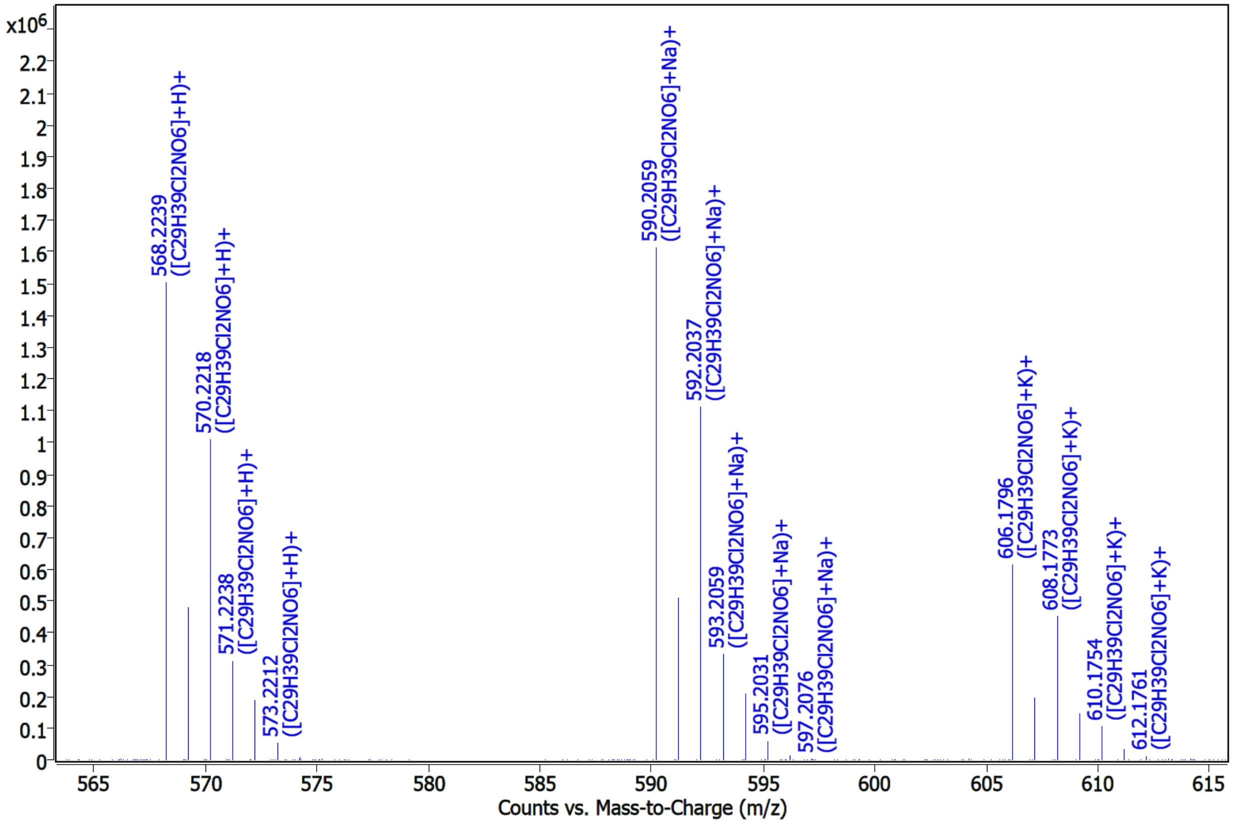

S8. ^{1}H NMR Spectrum ($CDCl_3$, 32 scans) of Compound **3**.

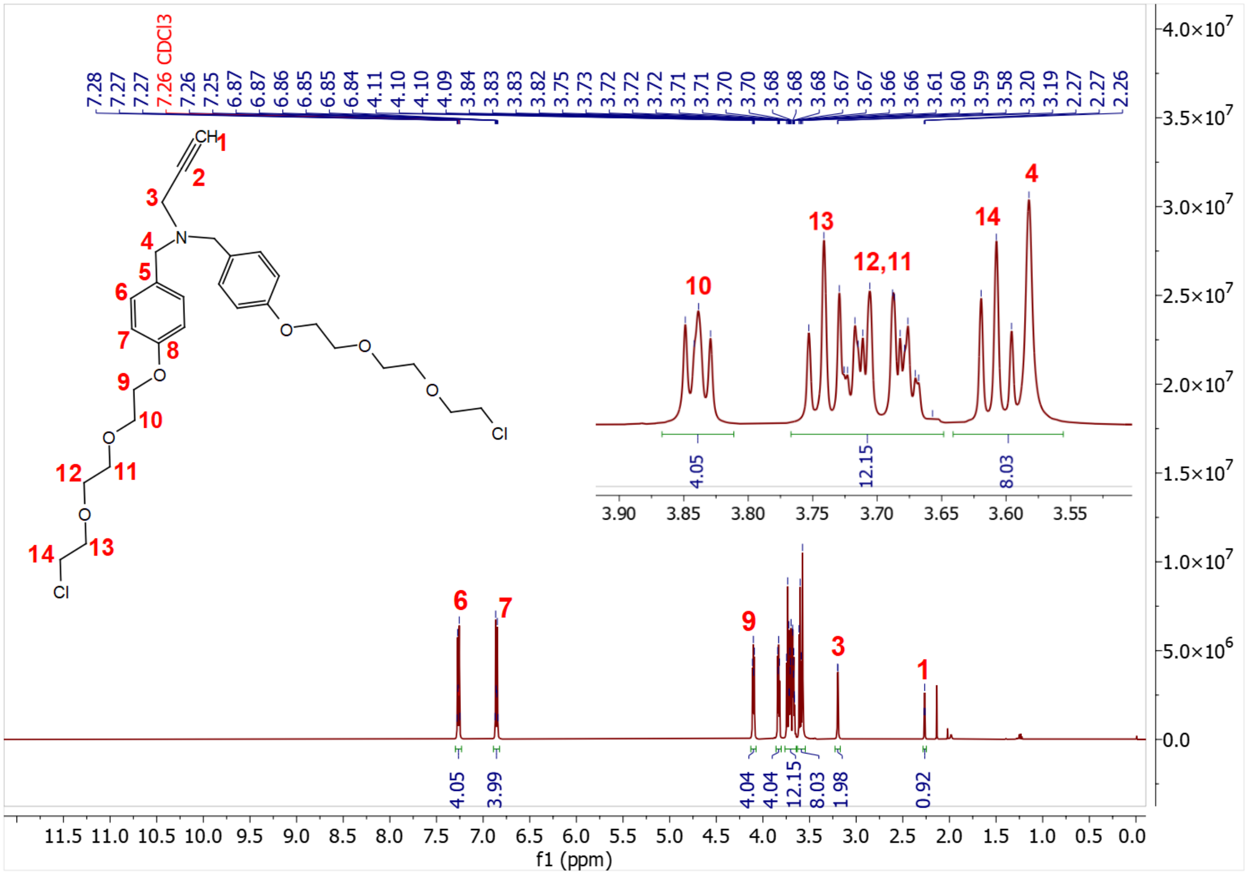

S9. ^{13}C NMR Spectrum ($CDCl_3$, 1024 scans) of Compound **3**.

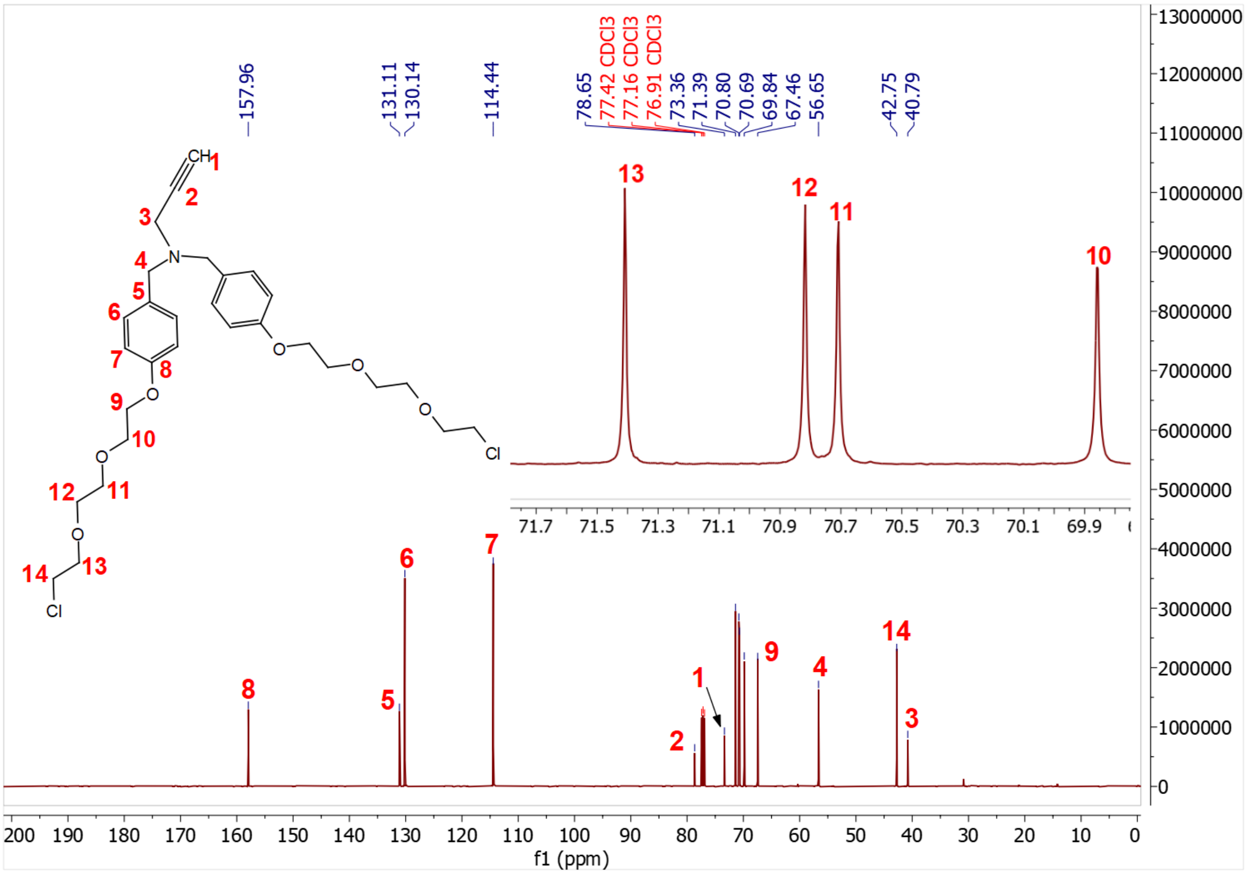

S10. MS Spectrum Results of Compound **4**.

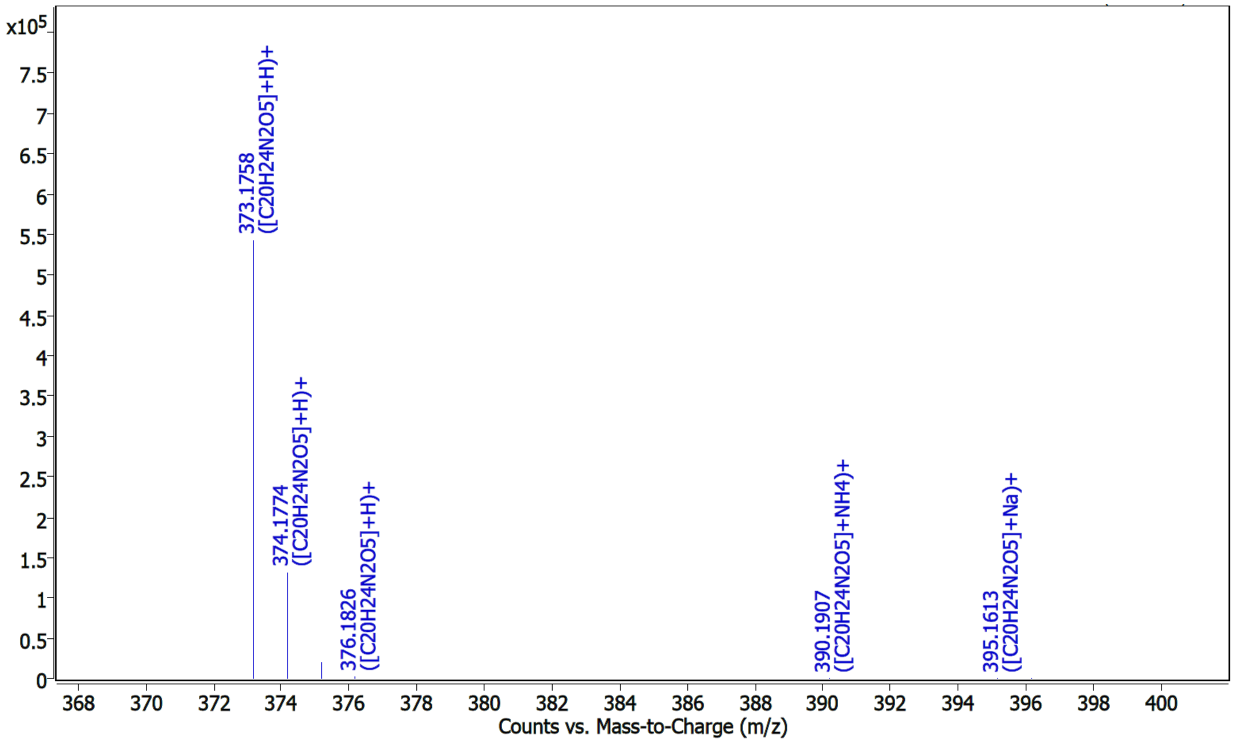

S11. ^{1}H NMR Spectrum ($CDCl_3$, 32 scans) of Compound **4**.

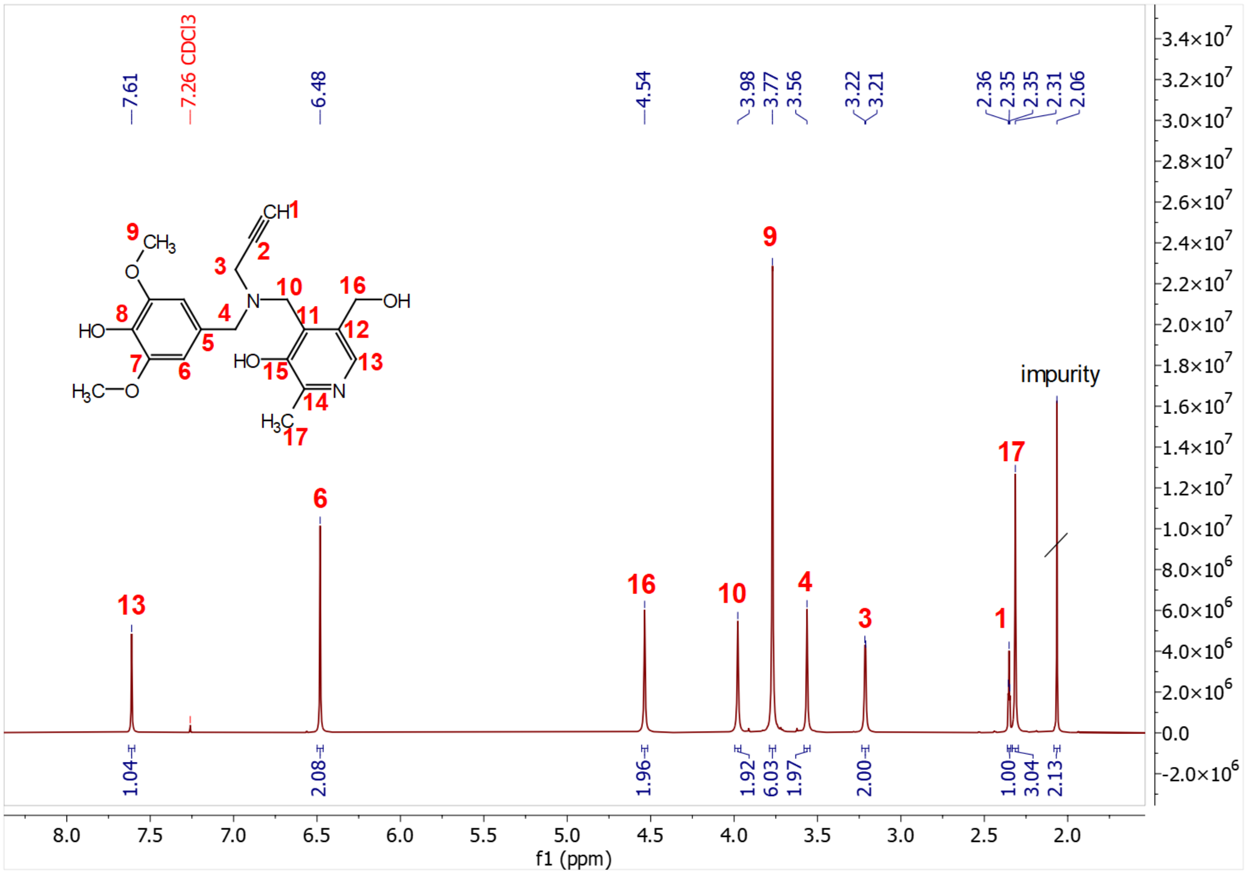

S12. ^{13}C NMR Spectrum ($CDCl_3$, 1024 scans) of Compound **4**.

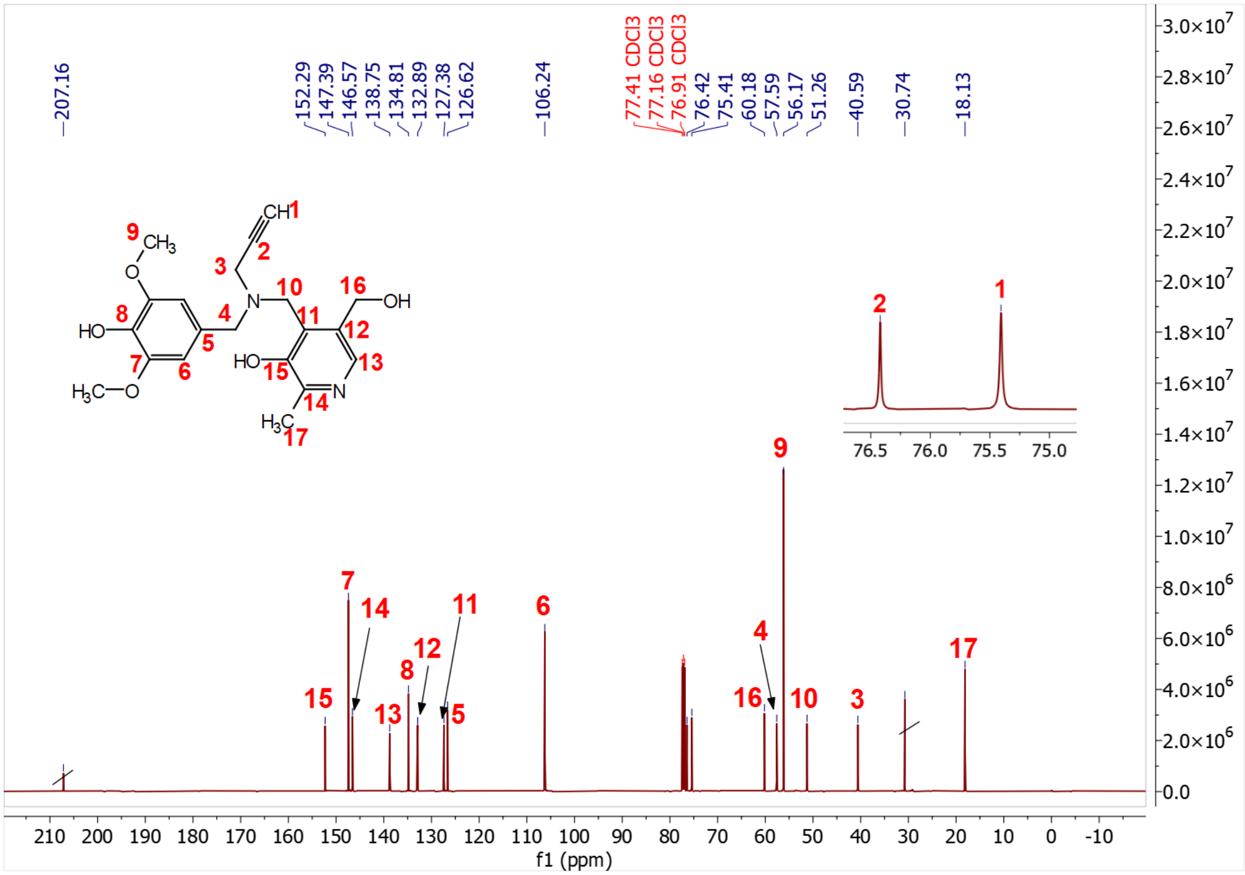

S13. MS Spectrum of Compound **6**.

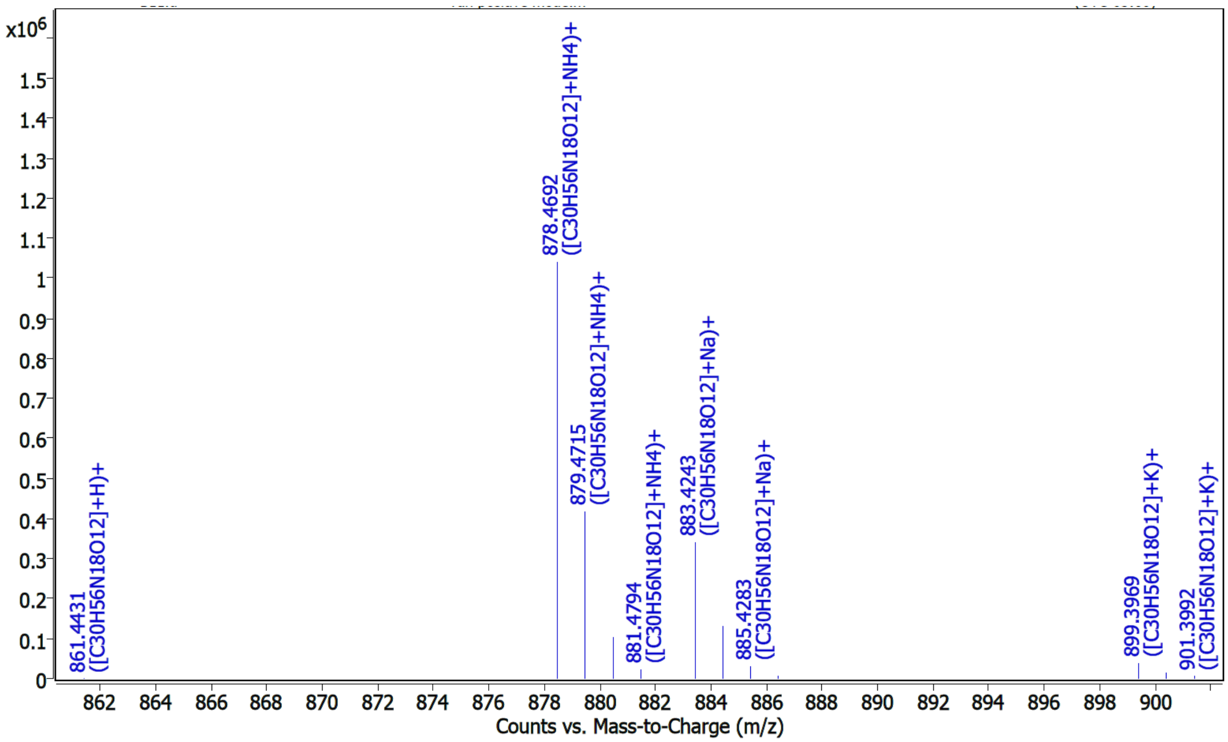

S14. ^{1}H NMR Spectrum (D-Acetone, 32 scans) of Compound **6**.

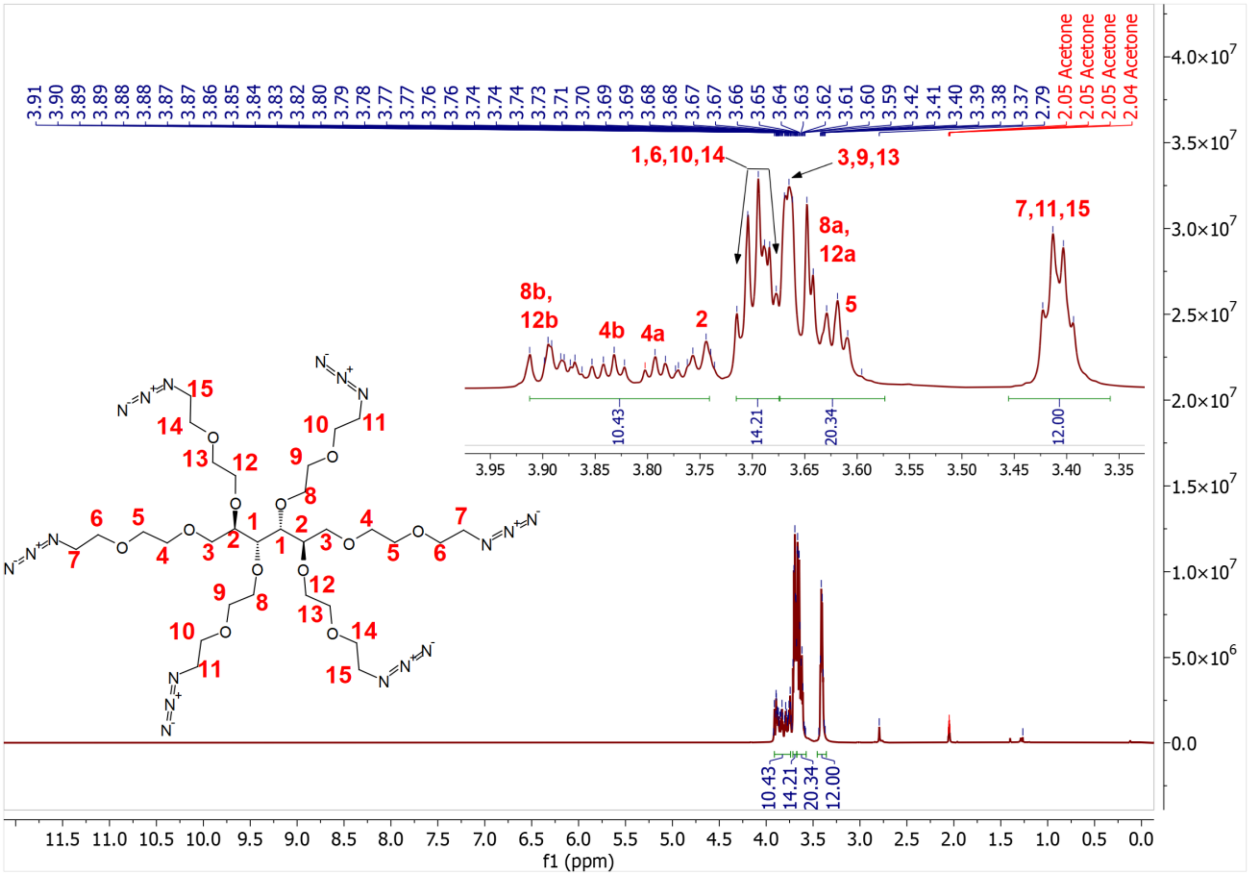

S15. ^{13}C NMR Spectrum (D-Acetone, 1024 scans) of Compound **6**.

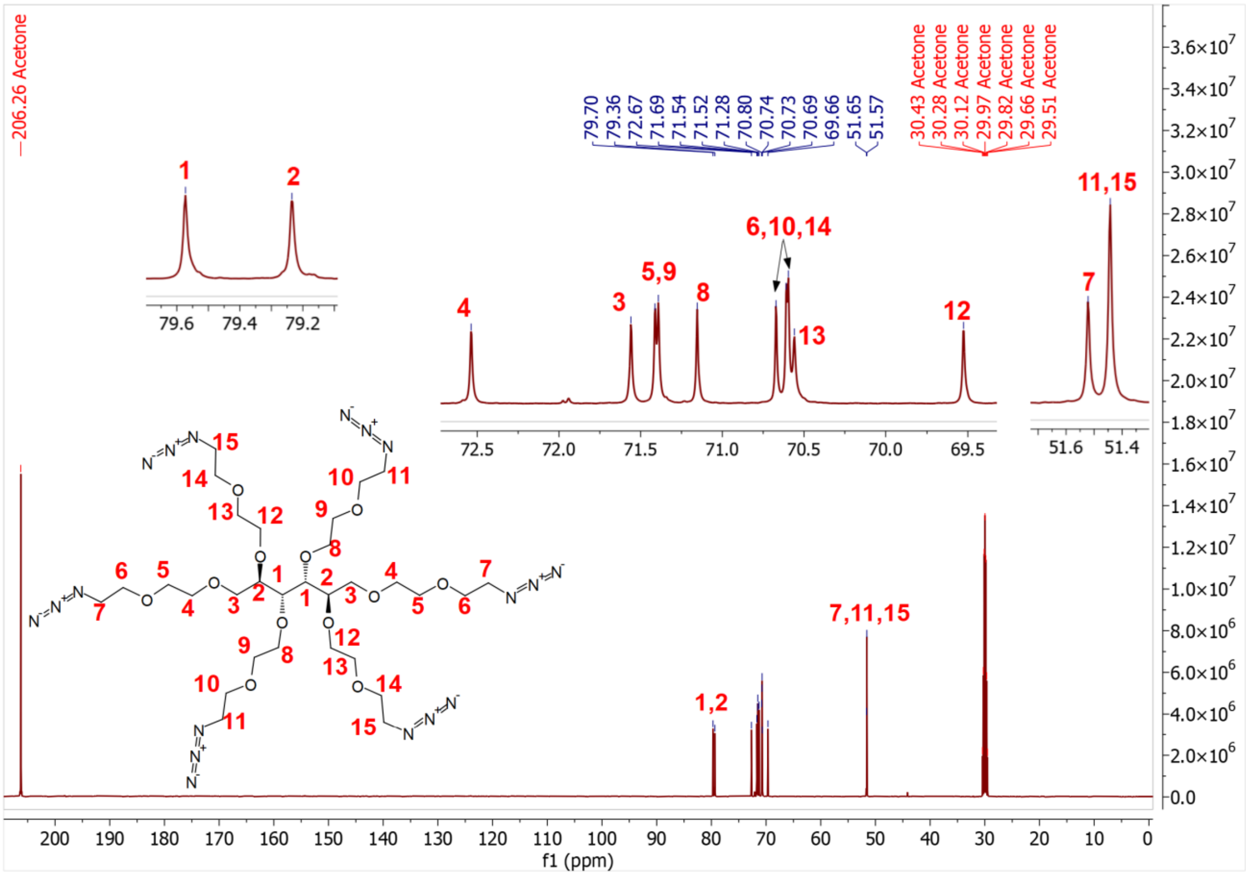

S16. MS Spectrum of Compound **7**.

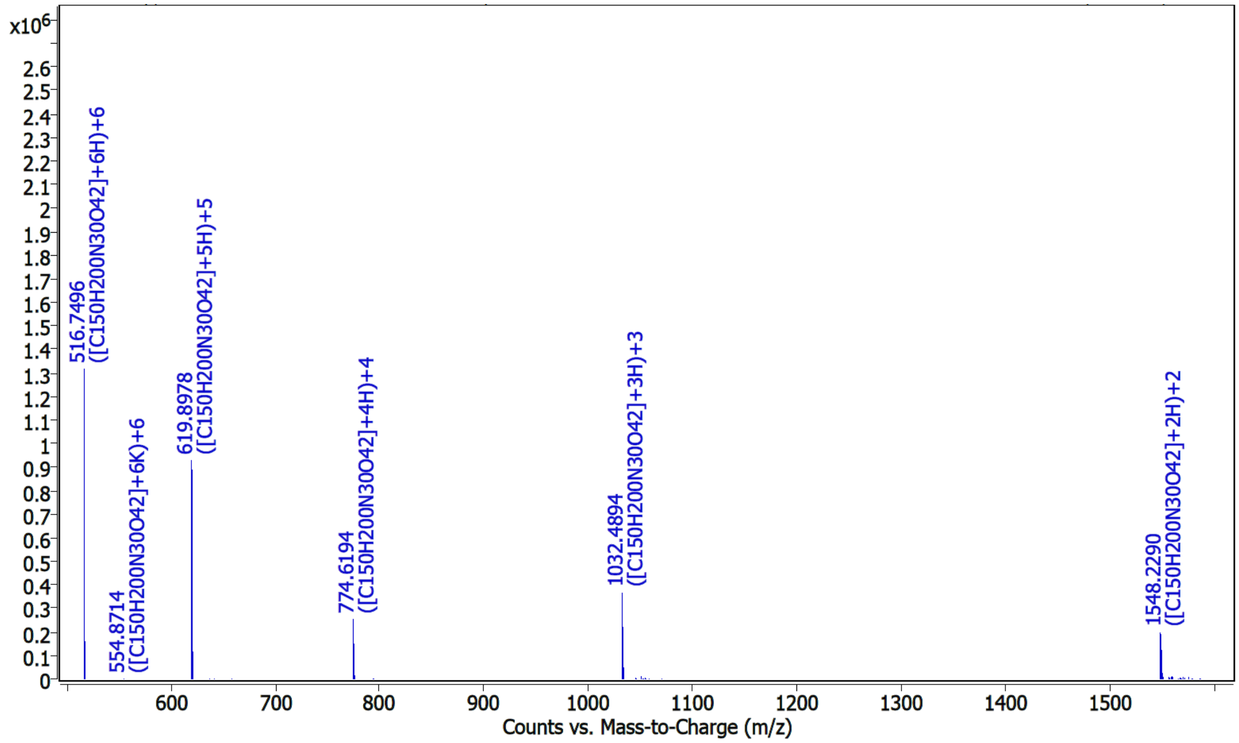

S17. ^{1}H NMR Spectrum (MeOD, 32 scans) of Compound **7**.

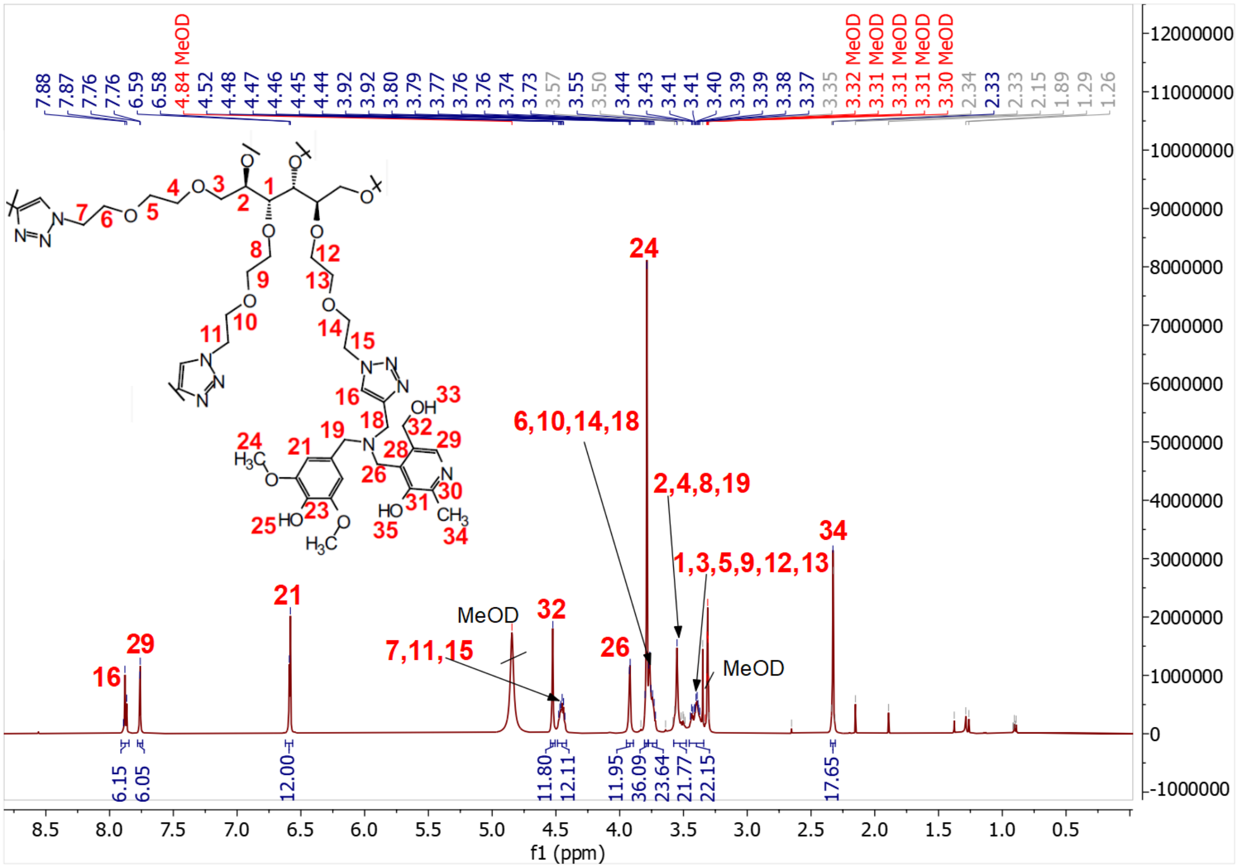

S18. ^{13}C NMR Spectrum (MeOD, 1024 scans) of Compound **7**.

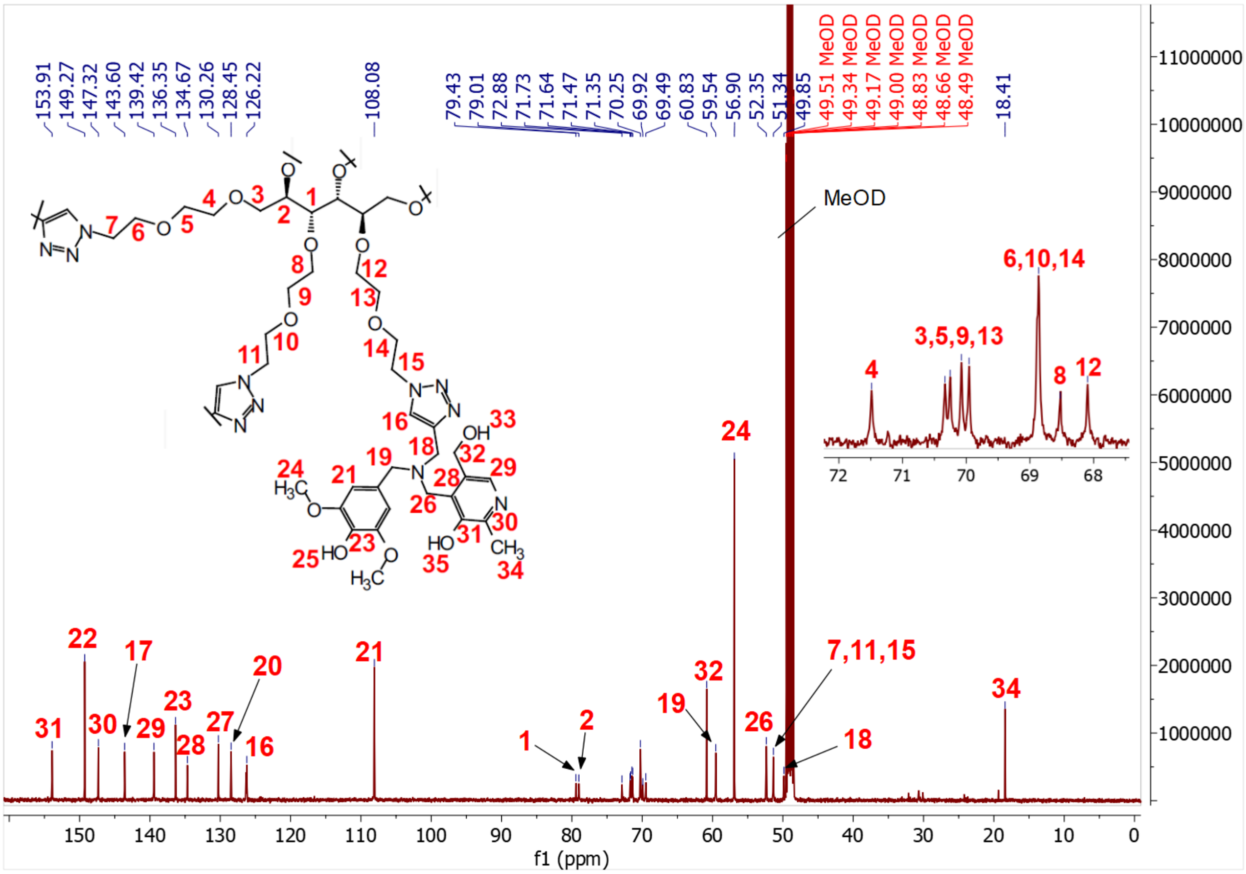

S19. MS Spectrum of Compound **8**.

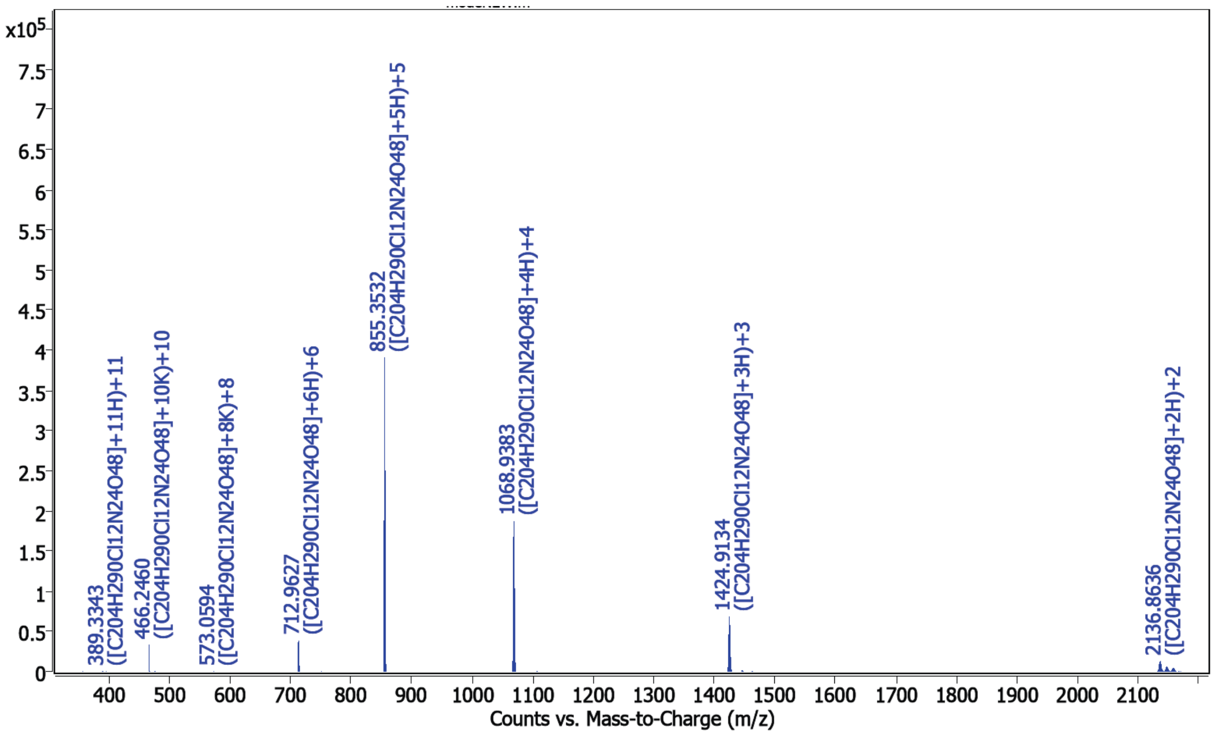

S20. ^{1}H NMR Spectrum ($CDCl_3$, 32 scans) of Compound **8**.

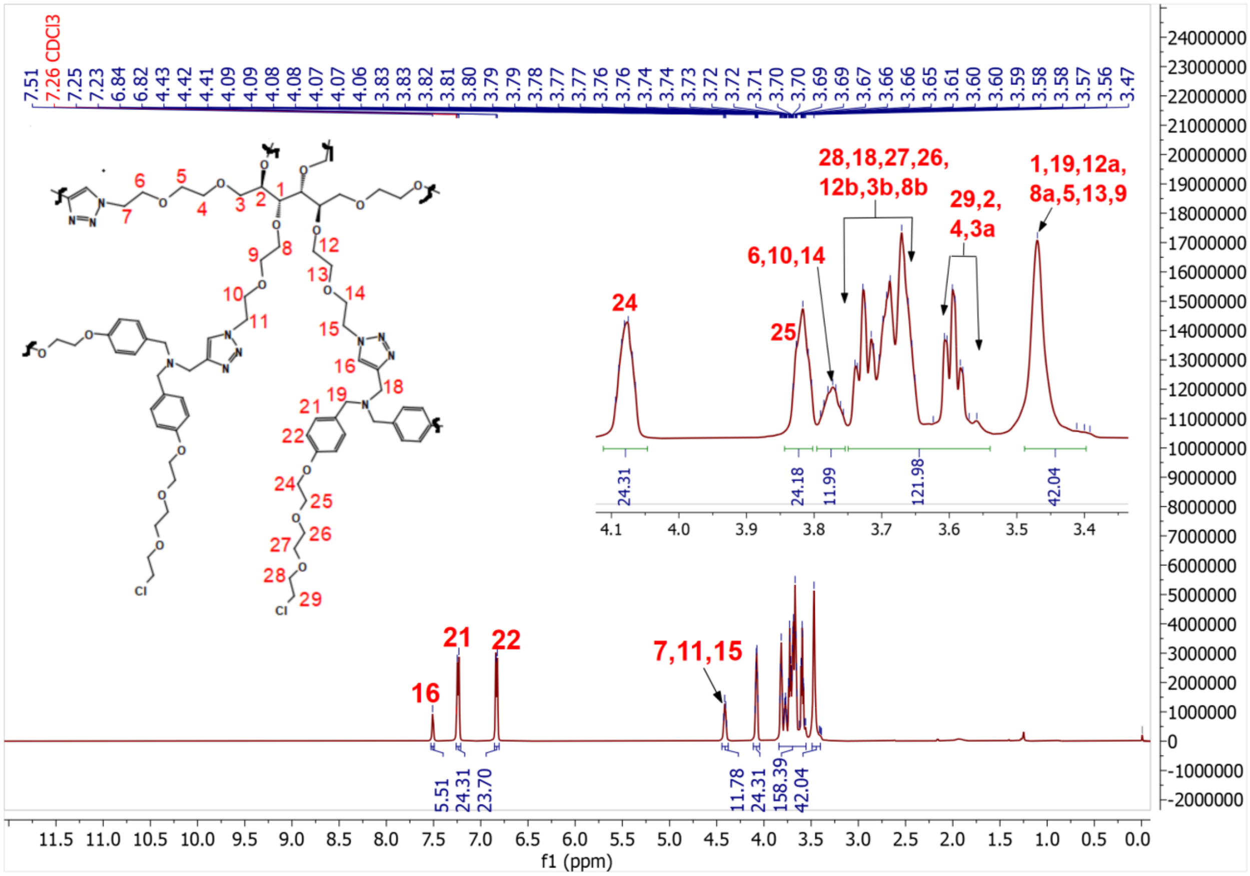

S21. ^{13}C NMR Spectrum ($CDCl_3$, 1024 scans) of Compound **8**.

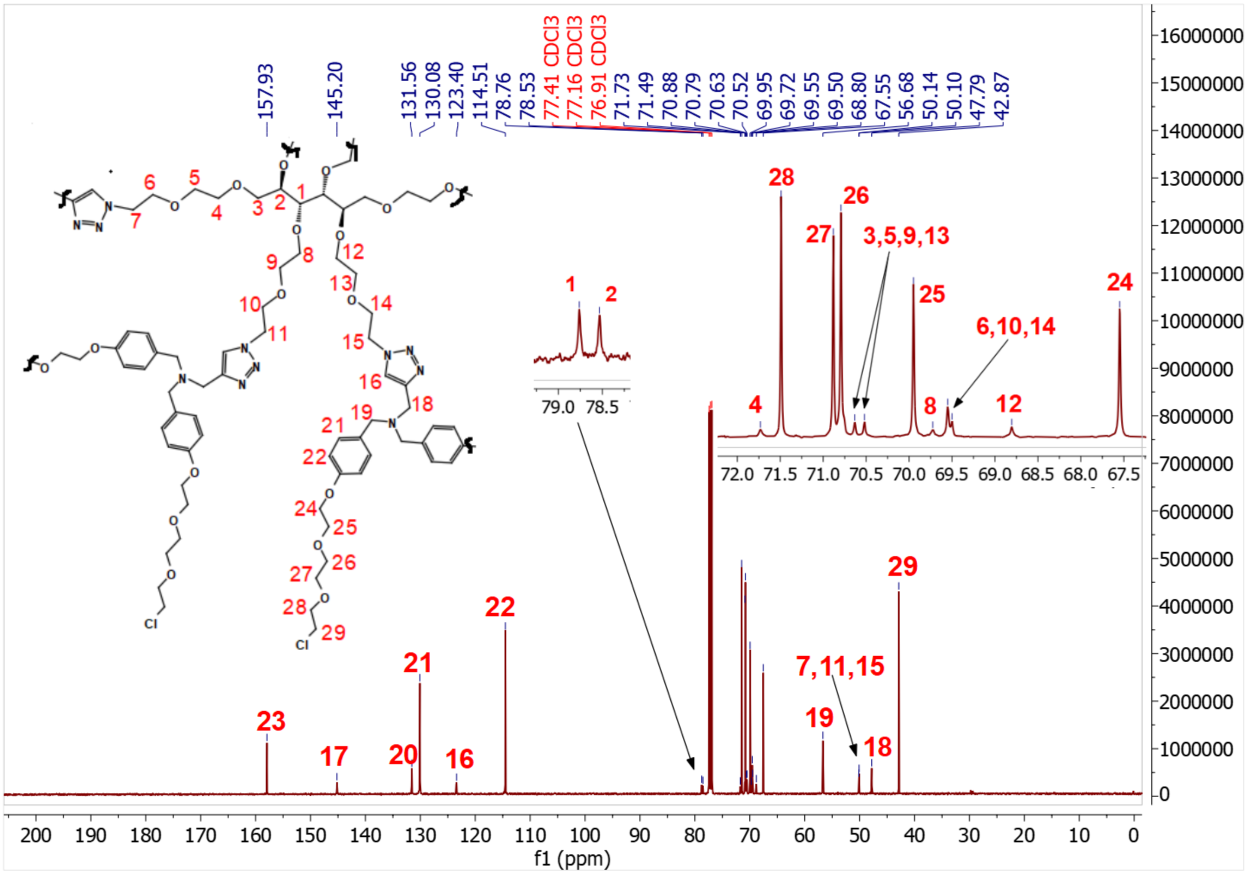

S22. MS Spectrum of Compound **9**.

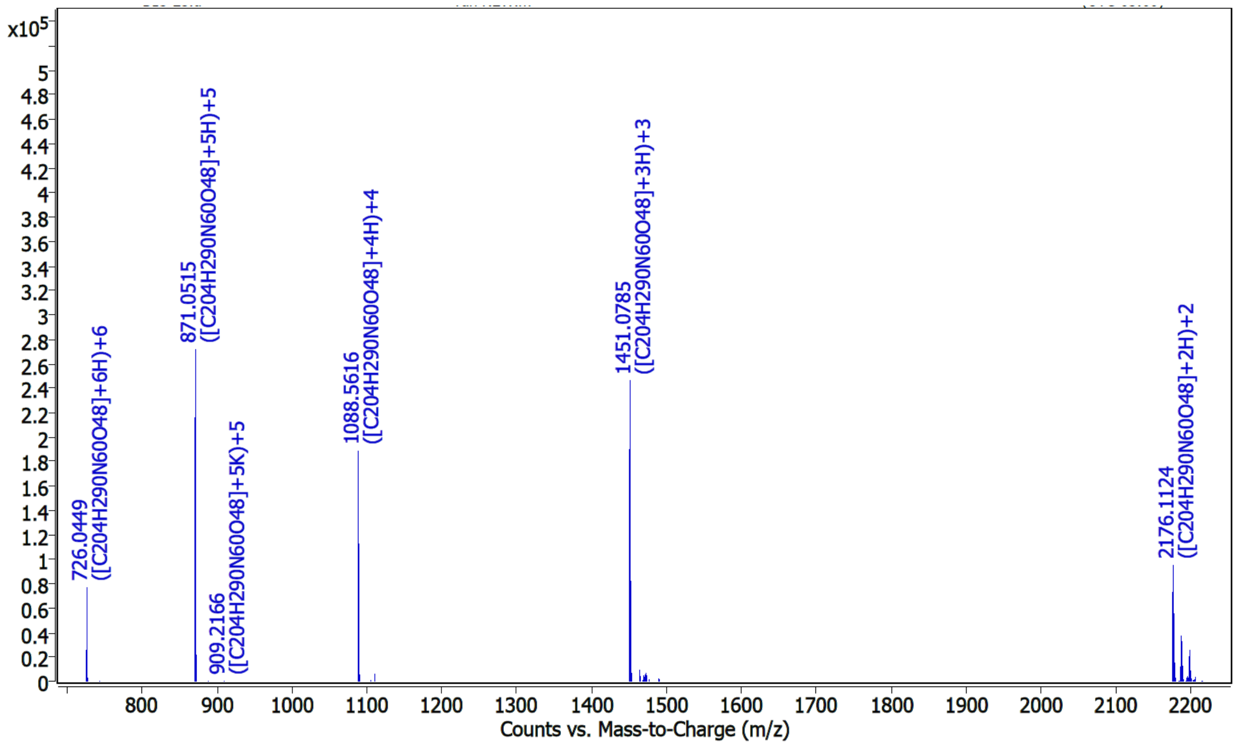

S23. ^{1}H NMR Spectrum (D-Acetone, 32 scans) of Compound **9**.

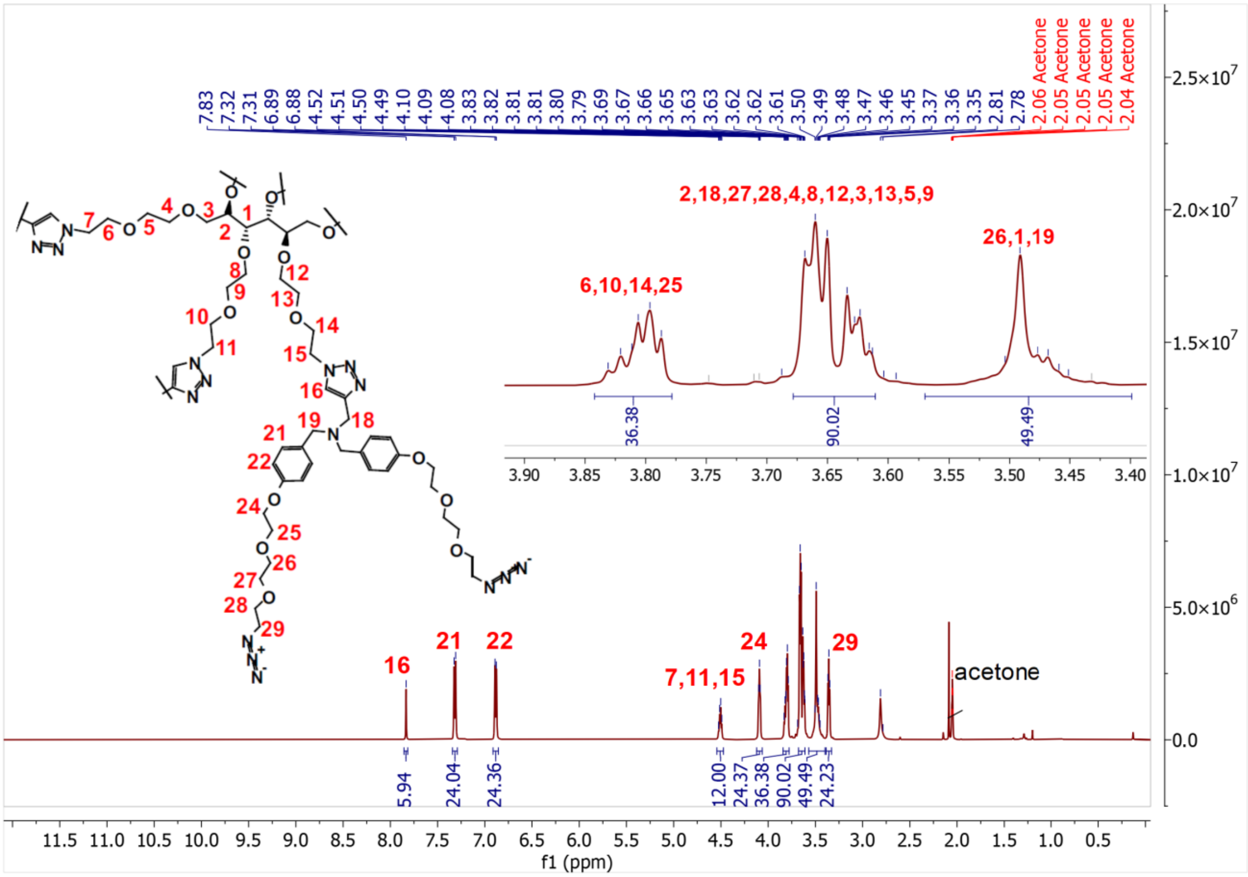

S24. ^{13}C NMR Spectrum (D-Acetone, 1024 scans) of Compound **9**.

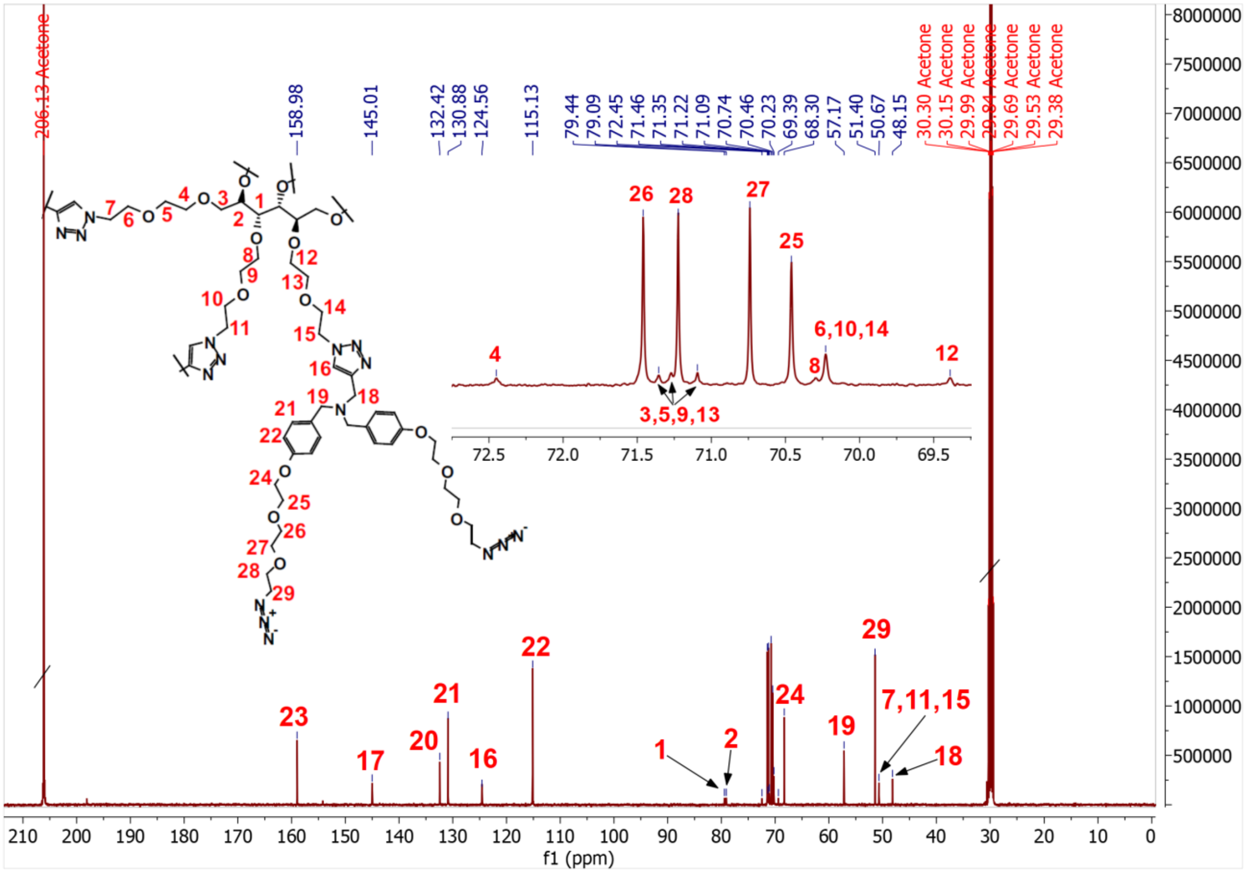

Supplement: Supplementary file 1 [file ap5c04280_si_001.pdf]
